# Rep Provides a Second Motor at the Replisome to Promote Duplication of Protein-Bound DNA

**DOI:** 10.1016/j.molcel.2009.11.009

**Published:** 2009-11-25

**Authors:** Colin P. Guy, John Atkinson, Milind K. Gupta, Akeel A. Mahdi, Emma J. Gwynn, Christian J. Rudolph, Peter B. Moon, Ingeborg C. van Knippenberg, Chris J. Cadman, Mark S. Dillingham, Robert G. Lloyd, Peter McGlynn

**Affiliations:** 1 School of Medical Sciences, Institute of Medical Sciences, University of Aberdeen, Foresterhill, Aberdeen AB25 2ZD, UK; 2 Institute of Genetics, University of Nottingham, Queen's Medical Centre, Nottingham NG7 2UH, UK; 3 DNA-Protein Interactions Unit, Department of Biochemistry, University of Bristol, Bristol BS8 1TD, UK

**Keywords:** DNA

## Abstract

Nucleoprotein complexes present challenges to genome stability by acting as potent blocks to replication. One attractive model of how such conflicts are resolved is direct targeting of blocked forks by helicases with the ability to displace the blocking protein-DNA complex. We show that Rep and UvrD each promote movement of *E. coli* replisomes blocked by nucleoprotein complexes in vitro, that such an activity is required to clear protein blocks (primarily transcription complexes) in vivo, and that a polarity of translocation opposite that of the replicative helicase is critical for this activity. However, these two helicases are not equivalent. Rep but not UvrD interacts physically and functionally with the replicative helicase. In contrast, UvrD likely provides a general means of protein-DNA complex turnover during replication, repair, and recombination. Rep and UvrD therefore provide two contrasting solutions as to how organisms may promote replication of protein-bound DNA.

## Introduction

Many barriers to replication fork progression exist within cells, all of which must be removed or bypassed for the completion of genome duplication. Failure to do so may lead to cell death, but blocked replisomes may also cause potentially catastrophic genome instability associated with cancer and many genetic disorders (Aguilera and Gómez-González, 2008). One class of potent blocks to replication are proteins bound to the template (Mirkin and Mirkin, 2007), with such collisions being numerous and unavoidable (Ivessa et al., 2003). Complexes associated with transcription present a particular problem, with direct collisions between replisomes and transcribing RNA polymerases (RNAPs) causing fork pausing in bacteria and eukaryotes (Azvolinsky et al., 2009; French, 1992). The importance of resolving these conflicts is supported by the ability of mutations within RNAP to suppress DNA repair defects in *E. coli* strains lacking the Holliday junction resolvase RuvABC (McGlynn and Lloyd, 2000). Suppression by these *rpo^∗^* mutations occurs by direct destabilization of RNAP elongation complexes, suggesting that RNAPs may be potent replicative blocks (Trautinger et al., 2005; Trautinger and Lloyd, 2002). The multiple mechanisms employed in all organisms to restart or displace stalled transcription complexes underline the importance of resolving such conflicts (Rudolph et al., 2007).

Recombination enzymes play important roles in processing of blocked replication forks. RecA is required for processing of forks blocked by UV-induced pyrimidine dimers in *E. coli* via a mechanism that requires the RecA loading factors RecFOR, RecJ exonuclease, and RecQ helicase (Courcelle et al., 2003). Induction of a nucleoprotein replication fork barrier, *RTS1*, in *S. pombe* also creates a requirement for recombination to maintain cell viability, suggesting that recombination-dependent processing of blocked forks is a general feature of genome duplication (Lambert et al., 2005). However, recombination provoked by *RTS1*-dependent replication blockage causes gross chromosomal rearrangements (Lambert et al., 2005). Hyperrecombination near Tus-*ter* and *lac* repressor-operator complexes in *E. coli* also indicates that genome instability is a general feature of recombination-dependent processing of blocked replisomes (Louarn et al., 1991; Vilette et al., 1992).

Alternatively, helicases could displace nucleoprotein complexes ahead of replisomes and so facilitate fork movement without the involvement of recombination, a possibility consistent with the displacement of proteins bound to DNA by many helicases and translocases (Yancey-Wrona and Matson, 1992). Bacteriophage T4 Dda helicase promotes movement of T4 replisomes along DNA bound by RNAPs in vitro (Bedinger et al., 1983), but Dda destabilizes transcription complexes independently of DNA replication (Liu et al., 1994). Thus, Dda may target transcription complexes rather than replication forks. However, *S. cerevisiae* cells lacking Rrm3p helicase have elevated pausing and breakage of replication forks at sites corresponding to nonhistone protein-DNA complexes, implicating Rrm3p as an accessory replicative helicase (Azvolinsky et al., 2009; Ivessa et al., 2003).

*E. coli* Rep and UvrD are helicases that translocate from 3′ to 5′ along ssDNA (Matson, 1986; Yarranton and Gefter, 1979). Although cells bearing single mutations in *rep* and *uvrD* are viable, *rep uvrD* cells are not (Taucher-Scholtz et al., 1983), while in cells lacking Rep, forks translocate at half the speed of those in wild-type (WT) cells (Lane and Denhardt, 1975). However, slower movement of forks in *rep* cells could reflect the function of Rep along with PriC in the reassembly of replisomes away from *oriC* (Heller and Marians, 2005), although lack of Rep-dependent replisome reloading is not responsible for *rep uvrD* lethality (Lestini and Michel, 2008). UvrD, unlike Rep, also has the ability to disassemble RecA nucleoprotein filaments in vitro (Veaute et al., 2005), as do *S. cerevisiae* Srs2 and human RTEL orthologs (Barber et al., 2008; Krejci et al., 2003). UvrD also counters toxic effects of RecQ-dependent recombination intermediates in vivo (Magner et al., 2007), while *rep uvrD* lethality can be suppressed by mutations in *recF*, *O*, *R*, *Q*, and *J* (Lestini and Michel, 2008; Petit and Ehrlich, 2002). UvrD might therefore function to inhibit formation of recombination intermediates at blocked forks (Magner et al., 2007).

Here, we demonstrate that Rep and UvrD promote movement of replisomes along protein-bound DNA regardless of the identity of the blocking nucleoprotein complex, that transcription complexes present the most significant of such blocks in vivo, and that accessory helicase function must be directed to the template strand opposite to that bound by the primary replicative helicase. Moreover, physical and functional interaction of Rep but not UvrD with the replicative helicase indicates that Rep is a component of the replisome. We conclude that Rep provides a fork-specific motor to facilitate replication of protein-bound template, whereas UvrD, highly abundant in vivo, provides a generalized motor for nucleoprotein complex turnover. Rep and UvrD therefore illustrate two solutions to the direct promotion of replisome movement along protein-bound DNA.

## Results

### Rep and UvrD Promote Replication of Protein-Bound DNA In Vitro

**Figure 1:**
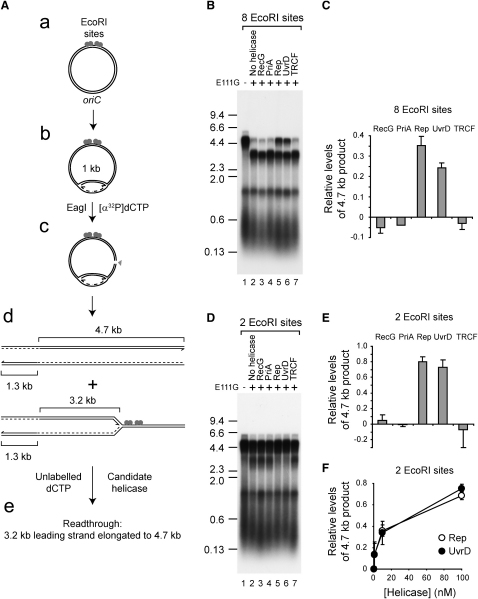
Rep and UvrD Promote Replication Fork Movement through EcoRI E111G-DNA Complexes In Vitro (A) Relative positions of *oriC* and EcoRI sites within plasmid templates, the site of cleavage by EagI, and the predicted sizes of leading strand products generated with and without replication blockage. (B) Denaturing agarose gel of replication products from pPM594 with and without E111G in the presence of the indicated helicases/translocases. (C) Levels of the 4.7 kb leading strand generated from pPM594 in the presence of E111G and the indicated enzymes relative to control reactions in lanes 1 and 2 in (B). (D) Replication products with pME101. (E) Levels of the 4.7 kb leading strand generated with pME101, plus E111G and the indicated enzymes relative to control reactions in lanes 1 and 2 in (D). (F) Relative levels of the 4.7 kb leading strand generated with pME101 plus E111G at increasing concentrations of Rep and UvrD. E111G was present at 200 nM dimers in all assays, while helicases/translocases were at 100 nM unless indicated otherwise. Error bars represent standard deviation of the mean.

To search for helicases that might promote fork movement through protein-DNA complexes, we analyzed movement of reconstituted *E. coli* replisomes along template DNA bound by a model protein-DNA replication block. EcoRI E111G binds to its recognition sequence but has greatly reduced cleavage activity (King et al., 1989) (see also Figure S2B, lanes 1–3). Replication of plasmids bearing *oriC* and two or eight EcoRI sites was initiated by addition of DnaA and replisome components followed by cleavage with EagI. Cleavage enabled passage of a single fork through the EcoRI sites to be monitored, since fork progression in the absence of a topoisomerase could occur only after relief of replication-induced positive supercoiling by restriction enzyme cleavage (Marians et al., 1998) (Figures 1Ab and 1Ac). In the absence of E111G, replication generated lagging strands of approximately 0.5 kb and leading strands of 4.7 and 1.3 kb (Figures 1B and 1D, lane 1). Upon addition of E111G, there was a decrease in the amount of the 4.7 kb leading strand, together with the appearance of a 3.2 kb product with a greater inhibitory effect observed for eight as opposed to two EcoRI sites (compare lanes 1 and 2 in Figures 1B and 1D; Figure S1). This 3.2 kb product was the size expected if clockwise-moving forks stopped at the E111G complexes (Figure 1Ad). Generation of this 3.2 kb leading strand also required EcoRI sites within the template DNA in addition to E111G, while levels of this truncated leading strand were dependent on E111G concentration (Figure S1). EcoRI E111G bound to its cognate DNA-binding site therefore provided a barrier to replisome movement.

The template bearing eight EcoRI sites was employed to provide an easily detectable signal for screening of *E. coli* enzymes that could relieve replisome inhibition. Helicases/translocases were chosen that have been implicated in processing of blocked forks or in displacement of proteins ahead of forks. Addition of Rep or UvrD after replisomes had become blocked resulted in increased levels of the 4.7 kb product, whereas RecG, PriA, and TRCF had no effect (Figures 1B and 1C). The same pattern was observed on the template bearing two rather than eight EcoRI sites, with eight out of ten blocked forks being able to complete replication of the template when Rep or UvrD were present (Figures 1D–1F). In contrast, RecG, PriA, and TRCF were not able to promote fork movement through this reduced nucleoprotein replicative barrier (Figures 1D and 1E). The relative increases in levels of the 4.7 kb product with Rep and UvrD were greater with two rather than eight EcoRI sites (compare Figures 1C and 1E), demonstrating that the ability of Rep and UvrD to relieve inhibition of replication was dependent on the number of E111G-DNA complexes.

Thus, Rep and UvrD promoted fork movement through E111G-DNA complexes, whereas RecG, PriA, and TRCF did not. Rep and UvrD both translocate 3′-5′ along ssDNA rather than dsDNA (Matson, 1986; Yarranton and Gefter, 1979), implying that fork movement was not promoted by translocation of Rep or UvrD along dsDNA ahead of the fork. Indeed, neither Rep nor UvrD displaced E111G from linearized, unreplicated DNA, as judged by their inability to relieve E111G-dependent inhibition of DNA cleavage by a catalytically competent EcoRI (Figure S2). Promotion of replisome movement along protein-bound DNA by Rep and UvrD therefore did not occur by indirect action along dsDNA remote from the fork. We conclude that the replication fork itself was required to provide ssDNA for Rep/UvrD binding and subsequent translocation.

### *rep uvrD* Lethality Is Dependent on Growth Conditions

**Figure 2:**
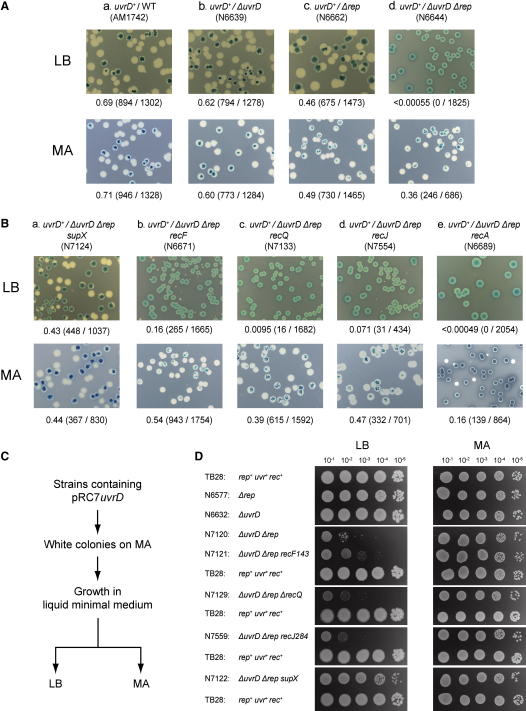
Analysis of Δ*rep* Δ*uvrD* Lethality (A) Retention or loss of pAM407 (pRC7*uvrD*) in strains bearing mutations in *rep* and/or *uvrD* as judged by blue/white colony color on LB (top row) and minimal agar (bottom row) containing X-gal and IPTG. Fractions of white colonies are indicated below each image with the actual number of white colonies and total colonies shown in parentheses. (B) Retention or loss of pAM407 from Δ*rep* Δ*uvrD* strains bearing a suppressor mutation identified subsequently as *rpoB*[T3713C], encoding RNAP L1238P (a); *recF* (b); *recQ* (c); *recJ* (d); and *recA* (e). Note that the suppressor strain N7124 in subpanel (a) was N7122, isolated as described in the text, into which pAM407 was reintroduced. (C) Strategy for the analysis of rich medium viability of plasmidless strains isolated on minimal agar. (D) The plasmid-free strains indicated were grown in liquid minimal medium before dilution and spotting onto LB or minimal agar.

We analyzed whether *rep uvrD* lethality (Taucher-Scholtz et al., 1983) was linked to promotion of replication through nucleoprotein complexes by Rep and UvrD. The basis of this lethality was probed using a plasmid loss assay employing a very low-copy, highly unstable plasmid bearing *lacIZYA* (pRC7), whose retention or loss in the absence of antibiotic selection can be monitored by blue/white colony color in strains harboring chromosomal Δ*lacIZYA* (Bernhardt and de Boer, 2004). Maintenance of pRC7 derivatives expressing *uvrD* or *rep* was studied in *rep^+^ uvrD^+^*, single-mutant and double-mutant strains. On rich growth medium, pRC7-encoded UvrD could be lost with high frequency in *rep*^+^ *uvrD*^+^ and in strains bearing single mutations in either gene, but no plasmidless colonies were detected in a Δ*rep* Δ*uvrD* strain, as expected (Figures 2Aa–2Ad, top row). In contrast, on minimal medium, plasmidless colonies formed regardless of the presence or absence of *rep* and *uvrD* (Figures 2Aa–2Ad, bottom row). When plasmidless segregants of Δ*rep* Δ*uvrD* obtained on minimal agar were cultured in liquid minimal medium and then plated onto Luria-Bertani medium (LB), there was a large decrease in colony-forming ability as compared with Δ*rep* or Δ*uvrD* strains, a viability defect not seen when the same liquid minimal medium cultures were plated onto minimal agar (Figures 2C and 2D, compare N6577, N6632, and N7120). The original plasmidless colonies obtained upon plating *uvrD^+^*/Δ*rep* Δ*uvrD* onto minimal agar did not, therefore, contain suppressor mutations that alleviated Δ*rep* Δ*uvrD* inviability. We conclude that absence of Rep and UvrD is lethal under rich but not minimal medium growth conditions.

### Suppression of *rep uvrD* Rich Medium Lethality

**Figure 3:**
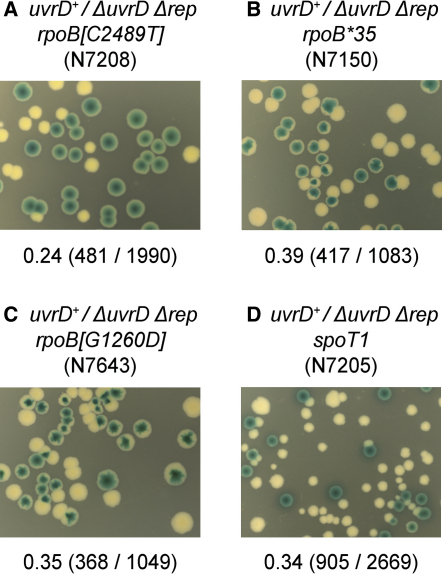
Suppression of Δ*rep* Δ*uvrD* Lethality by Mutations that Destabilize Transcription Complexes (A–D) Retention or loss of pAM407 in Δ*rep* Δ*uvrD* strains bearing mutations in *rpoB* (A, B, and C) or *spoT* (D) on LB/X-gal/IPTG. Note that *rpoB*[C2489T] was isolated in this study, as described in the text, as N7181. N7208 was formed by reintroduction of pAM407 into N7181.

Plating of plasmidless segregants of Δ*rep* Δ*uvrD* grown in liquid minimal medium onto LB agar did generate a few large colonies at low dilutions, indicating that effective suppression of rich medium lethality could occur (Figure 2D, N7120). Nine independent suppressors of Δ*rep* Δ*uvrD* rich medium lethality were isolated using this approach. These suppressors conferred on Δ*rep* Δ*uvrD* cells plating efficiencies on LB similar to those seen with *rep*^+^ *uvrD*^+^ (Figure 2D, compare N7122 with TB28). Reintroduction of pRC7 encoding UvrD into each Δ*rep* Δ*uvrD* suppressor strain also revealed that large plasmid-free colonies could now form with high frequency on LB (compare Figure 2Ad with 2Ba; Figure 3A) (data not shown). Thus, mutations could arise that restored the viability of Δ*rep* Δ*uvrD* cells on rich medium.

Null mutations in *recF*, *O*, *R*, *Q*, and *J* suppress Δ*rep* Δ*uvrD* lethality (Lestini and Michel, 2008; Petit and Ehrlich, 2002). The ability of *uvrD*^+^/Δ*rep* Δ*uvrD* strains bearing mutations in *recF*, *Q*, or *J* to form plasmidless segregants was compared, therefore, with the suppressors isolated above. *recF*, *Q*, and *J* mutations did allow formation of plasmidless colonies on LB, but these arose with reduced frequencies and were significantly smaller when compared with those obtained from *rep*^+^ *uvrD*^+^ *rec*^+^ cells or from the above nine suppressors of Δ*rep* Δ*uvrD* rich medium lethality (compare Figures 2Bb–2Bd with 2Aa and 2Ba) (data not shown). However, large plasmidless Δ*rep* Δ*uvrD recF*/*Q*/*J* colonies were obtained at high frequency on minimal agar, as seen with Δ*rep* Δ*uvrD rec*^+^ (compare Figures 2Bb–2Bd with 2Ad, bottom row). Plasmidless segregants were, as before, grown in liquid minimal medium and then plated onto both minimal and LB agar. Δ*rep* Δ*uvrD recF*, *Q*, and *J* strains formed very small colonies at reduced frequencies on LB agar as compared with the suppressors isolated above (Figure 2D, compare N7121, N7129, and N7559 with TB28 and N7122; data not shown). In contrast, sizes and frequencies of Δ*rep* Δ*uvrD recF*/*Q*/*J* colonies on minimal agar were indistinguishable from all other tested strains (Figure 2D).

We conclude that lack of RecF, Q, or J provides very limited suppression of Δ*rep* Δ*uvrD* rich medium lethality, in contrast to the high-level suppression found in the suppressors isolated in this study. RecFORQJ-dependent formation of RecA nucleoprotein filaments is not, therefore, the primary cause of rich medium lethality in Δ*rep* Δ*uvrD* cells.

### *rep uvrD* Cells Are Hypersensitive to Nucleoprotein Barriers to Replication

We sought to identify the suppressor mutations, isolated above, that restored Δ*rep* Δ*uvrD* rich medium viability. These suppressors were WT for *recF*, *recO*, *recR*, *dnaC*, and *ssb*, as shown by DNA sequencing, while cotransduction analyses excluded mutations in the RNAP genes *rpoA* and *rpoC* (data not shown). Inactivation of either *recQ* or *recJ* could also be excluded based on the levels of suppression seen in Figures 2B and 2D. Similarly, plasmidless segregants of *uvrD*^+^/Δ*rep* Δ*uvrD recA* were not detected on LB, as expected (Petit and Ehrlich, 2002), although some were observed on minimal medium (Figure 2Be). Seven of these suppressors remain unidentified, but two of the suppressed Δ*rep* Δ*uvrD* strains contained mutations in *rpoB*, encoding the β subunit of RNAP, as determined by sequencing. N7122 (Figure 2D) harbored *rpoB*[T3713C], encoding a L1238P mutation, while N7181 bore *rpoB*[C2489T], encoding T830I (Figure 3A).

Both L1238 and T830 lie near to the DNA-binding channel in RNAP (Vassylyev et al., 2007). Suppression of the DNA repair, chromosome segregation, and cell division defects in strains lacking the Holliday junction resolvase RuvABC or the dsDNA end-specific helicase/exonuclease RecBCD can be effected by a class of RNAP mutations termed *rpo^∗^*, most of which also lie adjacent to the path of DNA through transcribing RNAP (McGlynn and Lloyd, 2000; Trautinger and Lloyd, 2002). Suppression via *rpo^∗^* correlates with destabilization of transcription complexes, demonstrating that conflicts between replication and transcription pose major barriers to cell viability (Trautinger et al., 2005). We tested, therefore, whether a well-characterized *rpo^∗^* mutation shown to destabilize stalled transcription complexes in vitro, *rpoB^∗^35* encoding H1244Q (Trautinger et al., 2005), could suppress the rich medium lethality of Δ*rep* Δ*uvrD*. *uvrD*^+^/Δ*rep* Δ*uvrD rpoB^∗^35* formed large, plasmidless colonies at high frequency on LB, demonstrating that *rpoB^∗^35* provided very effective suppression of Δ*rep* Δ*uvrD* lethality (Figure 3B). A second *rpo^∗^* mutation, encoding *rpoB*[G1260D] (Trautinger and Lloyd, 2002), provided a similar level of Δ*rep* Δ*uvrD* suppression (Figure 3C).

The stringent response regulator ppGpp, like *rpoB^∗^35*, destabilizes transcription complexes (Potrykus and Cashel, 2008; Trautinger et al., 2005), while a mutation that increases ppGpp levels 10-fold, *spoT1*, also suppresses DNA repair defects of *ruv* and *recB* strains (McGlynn and Lloyd, 2000; Trautinger and Lloyd, 2002). *uvrD*^+^/Δ*rep* Δ*uvrD spoT1* cells formed large plasmid-free colonies on LB, demonstrating that enhanced ppGpp levels suppressed the viability defects of Δ*rep* Δ*uvrD* (Figure 3D).

Methods of strain construction used in analysis of *rpoB^∗^35*, *rpoB[G1260D]*, and *spoT1* (Table S1) demonstrate that suppression is a direct consequence of these mutations. The decrease in transcription complex stabilities in vitro shown by both the *rpoB^∗^35*-encoded RNAP and by elevated ppGpp (caused by *spoT1*), together with the strong correlation between this destabilization and suppression of multiple genome stability defects in vivo, have been characterized extensively (Potrykus and Cashel, 2008; Trautinger et al., 2005; Trautinger and Lloyd, 2002). Suppression of Δ*rep* Δ*uvrD* lethality by *rpoB^∗^35* and *spoT1* indicates, therefore, that suppression may occur directly via destabilization of transcription complexes and that this destabilization reduces the known barriers to replication posed by direct collision between replisomes and transcribing RNAPs (Azvolinsky et al., 2009; French, 1992; Mirkin and Mirkin, 2007; Rudolph et al., 2007; Trautinger et al., 2005). However, suppression by *rpo* or *spoT1* mutations could conceivably occur indirectly via mechanisms unrelated to the lowering of replicative barriers posed directly by transcribing RNAPs. RNAP mutations and elevation of ppGpp levels may alter global patterns of transcription, increasing or decreasing levels of specific gene products (Potrykus and Cashel, 2008). Alternatively, Rep and/or UvrD could conceivably have uncharacterized roles in the maintenance of transcription, such as removal of protein roadblocks out of the path of transcribing RNAPs rather than out of the path of replisomes.

A corollary of direct suppression by lowering of transcription complex barriers to replication is that lack of Rep and UvrD reduces the capacity of replisomes to move through protein-DNA complexes, while *rpoB^∗^35* compensates by reducing the stability of a major class of protein-DNA complexes (transcription complexes) encountered by replisomes. In contrast, suppression by altered patterns of gene expression or by bypass of Rep/UvrD function in transcription would not entail any reduced ability of replisomes to move along protein-bound DNA. We tested, therefore, whether Δ*rep* Δ*uvrD rpoB^∗^35* cells had a reduced ability to tolerate nucleoprotein complexes as compared with *rep^+^ uvrD^+^ rpoB^∗^35* cells or cells lacking only one helicase. In other words, could the effects of *rpoB^∗^35* on Δ*rep* Δ*uvrD* cells be reversed by induction of a stable nucleoprotein replicative barrier?

**Figure 4:**
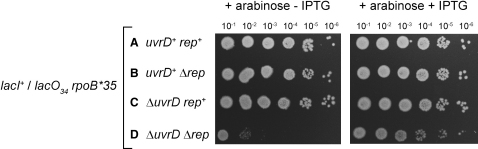
Suppressed Δ*rep* Δ*uvrD* Cells Remain Hypersensitive to an Artificial Nucleoprotein Barrier to Replication (A–D) Strains PM462–465 bearing 34 chromosomal *lac* operators plus pPM306, a plasmid bearing *lacI* under the control of an arabinose-inducible promoter, were tested for colony-forming ability upon expression of *lac* repressor. Cells were grown in LB in the absence of arabinose, and then serial dilutions were spotted onto LB agar containing arabinose without and with IPTG.

Thirty-four chromosomal tandem *lac* repressor-operator complexes provide an inducible replicative barrier that is tolerated in otherwise WT cells and in cells lacking either Rep or UvrD (Payne et al., 2006). *lacO_34_* was introduced into *rpoB^∗^35* strains, and the consequences of repressor expression on colony-forming ability were evaluated in the presence or absence of isopropyl-β-D-thiogalactopyranoside (IPTG). Expression of repressor had no detectable effect on viability of *rep*^+^ *uvr^+^ rpoB^∗^35* or of strains bearing mutations in either helicase gene with or without IPTG (Figures 4A–4C). However, the colony-forming ability of Δ*rep* Δ*uvrD rpoB^∗^35* was reduced dramatically upon repressor expression, a reduction that was alleviated by IPTG (Figure 4D). Suppression by *rpoB^∗^35* was reversed, therefore, by the tandem repressor-operator complexes, demonstrating that cells lacking both Rep and UvrD have a reduced inherent capacity to tolerate stable nucleoprotein complexes. Thus, suppression by *rpoB^∗^35* does not occur via altered patterns of gene expression, nor by circumvention of direct roles of Rep or UvrD in transcription. Instead, these data strongly support a model in which *rpoB^∗^35* suppresses *Δrep ΔuvrD* lethality by direct reduction of the replicative barriers posed by transcription complexes.

We conclude that Rep and UvrD promote movement of forks through blocking protein-DNA complexes and that this promotion occurs regardless of the identity of the nucleoprotein complexes, but that in WT cells, transcribing RNAPs provide the most significant type of nucleoprotein barrier encountered by forks.

### Correlation of In Vitro Promotion of Fork Movement with Complementation of *rep uvrD* Lethality

**Figure 5:**
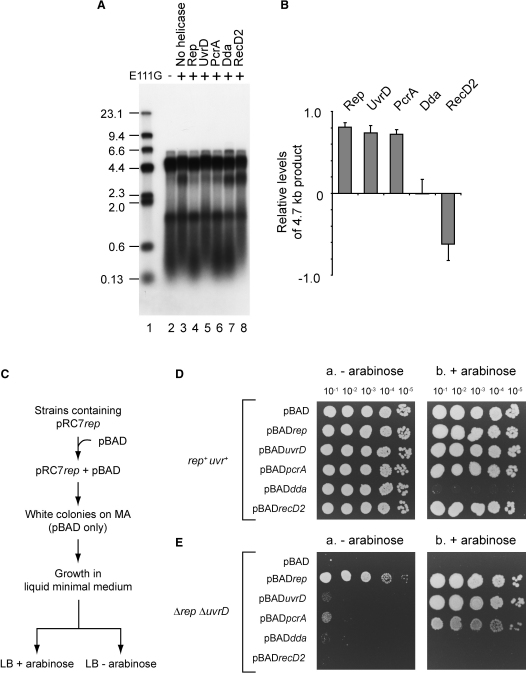
Correlation between Helicase-Mediated Promotion of Fork Movement In Vitro and Complementation of Δ*rep* Δ*uvrD* Lethality In Vivo (A) Denaturing agarose gel of replication products from pME101 (two EcoRI sites) with and without E111G (200 nM dimers) in the presence of the indicated helicases (100 nM). (B) Levels of the 4.7 kb leading strand in the presence of the indicated helicases relative to control reactions in lanes 2 and 3 in (A). Error bars represent standard deviation of the mean. (C) Scheme for generation of strains containing helicase genes under the control of an arabinose-inducible promoter. (D and E) Colony-forming ability of *rep*^+^*uvrD*^+^ (N6524) and Δ*rep* Δ*uvrD* (N6556) strains lacking pRC7*rep* but bearing the indicated plasmids after growth in liquid minimal medium, subsequent dilution, and spotting onto LB containing kanamycin ± arabinose.

Δ*rep* Δ*uvrD* lethality can be complemented by a Rep/UvrD homolog from *Bacillus*, PcrA (Petit et al., 1998). We found that PcrA, like Rep and UvrD, promoted movement of reconstituted *E. coli* replisomes through DNA-E111G complexes (Figures 5A and 5B). Rep, UvrD, and PcrA are all Superfamily 1 helicases that translocate 3′-5′ along ssDNA to effect duplex unwinding (Singleton et al., 2007). We tested whether Superfamily 1 helicases that move in the opposite direction along ssDNA also promote replisome movement along protein-bound DNA. However, neither bacteriophage T4 Dda (Jongeneel et al., 1984) nor *Deinococcus radiodurans* RecD2 (Wang and Julin, 2004) could promote movement of *E. coli* replisomes through E111G complexes in vitro (Figures 5A and 5B). Indeed, addition of RecD2 resulted in decreased production of the full-length 4.7 kb leading strand (Figure 5B), implying that RecD2 reduced readthrough of replisomes at DNA-E111G complexes. Thus, the ability of helicases to promote fork movement through protein-DNA complexes in vitro correlates with polarity of helicase translocation along ssDNA. This translocation polarity, 3′-5′, is opposite to that of the replicative helicase DnaB.

The ability of Dda and RecD2 to complement Δ*rep* Δ*uvrD* lethality was also tested. *rep*^+^ *uvrD*^+^ and Δ*rep* Δ*uvrD* strains containing plasmids bearing inducible helicase genes were generated on minimal medium and then plated onto rich medium ± arabinose (Figure 5C). As expected, high-level expression of *rep*, *uvrD*, or *pcrA* complemented Δ*rep* Δ*uvrD* lethality (Figures 5Db and 5Eb). Complementation of Δ*rep* Δ*uvrD* lethality was also observed with pBAD*rep* in the absence of arabinose, albeit with reduced colony size, indicating that low-level expression of *rep* but not *uvrD* or *pcrA* could maintain viability (Figure 5Ea). However, neither *dda* nor *recD2* expression promoted survival of Δ*rep* Δ*uvrD* cells (Figure 5E). Indeed, *dda* expression was toxic, as revealed in *rep*^+^ *uvrD*^+^ cells (compare Figures 5Da and 5Db).

Complementation of Δ*rep* Δ*uvrD* lethality in vivo was therefore observed only with helicases that also promoted replisome movement along protein-bound DNA in vitro. Moreover, complementation required high-level expression of *uvrD* (and *pcrA*) but not of *rep*.

### Rep Interacts with DnaB Physically and Functionally

**Figure 6:**
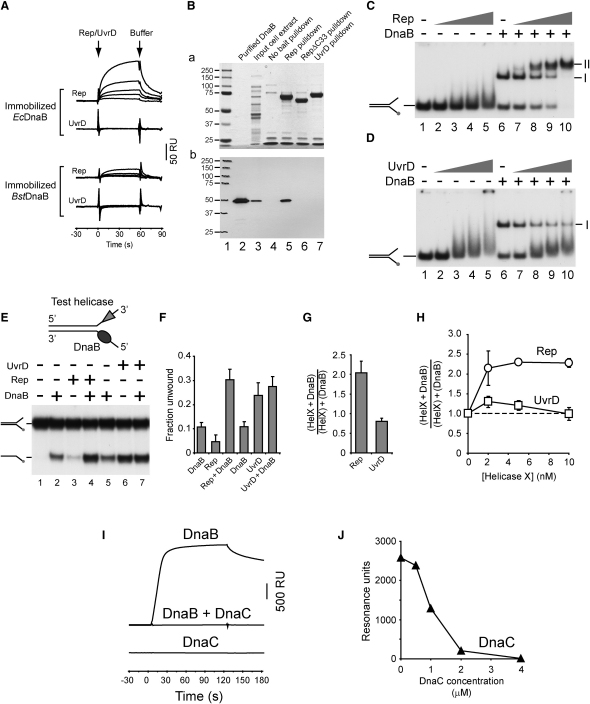
Rep Interacts with DnaB (A) Binding of Rep and UvrD to surface-immobilized *E. coli* and *B. stearothermophilus* DnaB (860 and 1705 resonance units, respectively), as measured by surface plasmon resonance. Concentrations of Rep and UvrD were 200, 500, 1000, 2000, and 4000 nM. (B) Coomassie-stained gel of pull-down assays from whole-cell extracts with biotinylated Rep, RepΔC33, and UvrD as bait (subpanel [a], lanes 5–7, respectively). Lanes 2 and 3 contained 100 ng of purified DnaB and 12 μg of untreated whole-cell extract, respectively, while protein from a mock pull-down experiment with no bait protein present was run in lane 4 (a). Subpanel (b) shows a western blot, using DnaB antibodies, of the pull-down assays shown in (a). (C) Binding of Rep and *E. coli* DnaB to forked DNA having two ssDNA arms. Concentrations of Rep were 1, 5, 10, and 25 nM, while DnaB was present at 100 nM hexamers as indicated. (D) Binding of UvrD and DnaB to forked DNA. Concentrations of UvrD were as for Rep in (C), and the concentration of DnaB was 100 nM hexamers. (E) Unwinding of forked DNA by *E. coli* DnaB (100 nM hexamers), Rep, and UvrD (both at 10 nM). (F) Fraction of the forked DNA substrate unwound by the indicated helicases. Protein concentrations were as in (E). Error bars represent standard deviation of the mean. (G) Relative levels of forked DNA unwinding by DnaB plus Rep/UvrD (“HelX”) in comparison to the sum of unwinding by each individual helicase. Protein concentrations were as in (E). (H) Relative levels of forked DNA unwinding by 100 nM DnaB hexamers in the presence of the indicated concentrations of Rep and UvrD. Concentrations >10 nM were not tested due to high levels of unwinding by UvrD. (I) Inhibition of the DnaB-Rep interaction by DnaC; binding of DnaB (1 μM monomers) and/or DnaC (4 μM monomers) to the C33^Rep^ peptide (260 resonance units surface-immobilized via an N-terminal biotin tag). (J) Interaction of DnaB with C33^Rep^ in the presence of increasing concentrations of DnaC. Conditions were otherwise as in (I).

The above data indicate that Rep and UvrD function at forks blocked by nucleoprotein complexes. We therefore screened for interactions between UvrD, Rep, and components of the replisome. No interactions with either UvrD or Rep were detected with primase; SSB; β sliding clamp; the DNA polymerase III αɛ; χψ or γ complexes; or θ, δ, δ′, χ, and γ subunits, as determined by surface plasmon resonance (data not shown). However, Rep did interact with the replicative helicase DnaB (Figure 6A). This interaction was specific. No interaction was detected between UvrD and DnaB, while there was greatly reduced interaction between Rep and *B. stearothermophilus* DnaB (Figure 6A). Moreover, using Rep and UvrD proteins as bait in pull-down experiments from *E. coli* whole-cell extracts, DnaB associated with Rep but not with UvrD (Figure 6B, compare lanes 5 and 7). Gel shift analyses indicated that while Rep in the absence of DnaB did not form a stable complex with a forked DNA substrate possessing both 3′ and 5′ ssDNA arms (Figure 6C, lanes 2–5), a specific DNA complex requiring both Rep and DnaB did form (Figure 6C, compare lanes 5, 6, and 10, complex II). In contrast, no detectable UvrD-DnaB-DNA complex was observed (Figure 6D). Thus, a UvrD-DnaB complex could not form either in the absence or the presence of DNA.

The same forked DNA substrate was used to analyze DNA unwinding by DnaB (a 5′-3′ translocase) in the absence and presence of Rep or UvrD (3′-5′ translocases) (Figure 6E). Enhanced unwinding was detected with DnaB + Rep but not with DnaB + UvrD, relative to levels of unwinding by the individual helicases (Figures 6F and 6G). High levels of cooperativity were detected at all tested concentrations of Rep, but not of UvrD (Figure 6H and data not shown). Enhanced unwinding was not, therefore, due simply to a lowering of the processivity barrier for Rep by limited unwinding by DnaB or vice versa, since such a generalized mechanism would have been applicable to UvrD also. Thus, Rep and DnaB interact functionally as well as physically.

We screened Rep mutants for their ability to interact with DnaB. The autoregulatory Rep 2B subdomain (Brendza et al., 2005) was not responsible for the Rep-DnaB interaction (Figure S3). We next tested Rep lacking the C-terminal 33 residues, RepΔC33. These residues are not conserved in UvrD and are disordered in Rep crystals (Korolev et al., 1997). No interaction between RepΔC33 and DnaB was observed, either by pull-down experiments from whole-cell extracts (Figure 6B, compare lanes 5 and 6) or by SPR (Figure S4). Furthermore, DnaB bound to a synthetic peptide corresponding to the C-terminal 33 residues of Rep (Figure S4), indicating that the Rep C terminus contacts DnaB directly. The affinity of this interaction was high, with the apparent K_D_ (equilibrium dissociation constant) of DnaB for Rep and C33^Rep^ being less than 100 nM, whereas that for RepΔC33 was 1700 nM (Figure S5).

**Figure 7:**
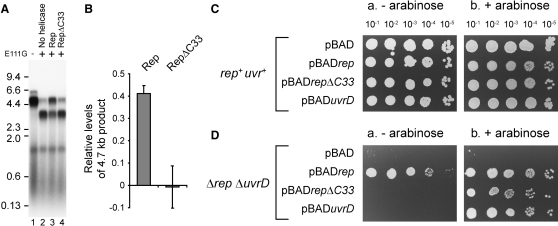
Promotion of Rep Function In Vitro and In Vivo by Interaction with DnaB (A) Denaturing agarose gel of replication products from pPM594 (eight EcoRI sites) with and without E111G (200 nM dimers) in the presence of Rep and RepΔC33 (100 nM). Note that RepΔC33 also failed to promote replisome movement along template bearing two EcoRI sites (data not shown). (B) Levels of the 4.7 kb leading strand in the presence of the indicated helicases relative to control reactions in lanes 1 and 2 in (A). Error bars represent standard deviation of the mean. (C and D) Colony-forming ability of *rep^+^ uvrD^+^* (N6524) and *Δrep ΔuvrD* (N6556) strains bearing the indicated plasmids, tested as described in Figure 5C.

RepΔC33 had a greatly reduced ability to promote movement of replisomes through DNA-E111G complexes in vitro (Figures 7A and 7B), even though RepΔC33 retained helicase activity (Figure S6). Moreover, unlike WT *rep*, a plasmid bearing *repΔC33* failed to complement *Δrep ΔuvrD* rich medium lethality in the absence of high-level expression of the helicase gene (Figure 7Da). High-level expression of *repΔC33* did complement to some extent, demonstrating that elevated concentrations of RepΔC33 were needed to compensate for lack of the Rep C terminus (Figure 7Db). Thus, the pattern of complementation by *repΔC33* resembled that of *uvrD* rather than WT *rep*. We conclude that interaction between the Rep C terminus and DnaB promotes Rep function in vitro and in vivo.

Interaction of Rep with DnaB implies that Rep is located at the replication fork. Fluorescent protein fusions to Rep, although complementing Δ*rep* Δ*uvrD* lethality, did not form detectable foci in vivo (data not shown) and so could not be used to probe the location of Rep. We therefore employed a second approach to probe the location of Rep. DnaB forms a stoichiometric complex with DnaC, a helicase loader that is essential for binding of DnaB to SSB-coated ssDNA during replisome assembly, but which dissociates from DnaB once DnaB is bound to ssDNA (Wahle et al., 1989). Thus, DnaB exists as a DnaB_6_.DnaC_6_ complex prior to replication initiation but is not bound by DnaC upon binding of DnaB to the chromosome. We tested the effect of DnaC upon the interaction of DnaB with the Rep C terminus and found that the interaction of DnaB with C33^Rep^ was severely inhibited in the presence of DnaC (Figures 6I and 6J). DnaC alone did not interact with the peptide, demonstrating that this inhibition was not due simply to competition between DnaB and DnaC for binding to the peptide (Figure 6I). We conclude that interaction of Rep with DnaB is inhibited within the context of a DnaB_6_.DnaC_6_ complex and that binding of Rep and DnaB occurs only after loading of DnaB onto the chromosome.

## Discussion

We have discovered that cells lacking Rep and UvrD die primarily because of hypersensitivity to nucleoprotein replicative blocks, that this hypersensitivity correlates with the ability of either helicase to resuscitate replisomes blocked by protein-DNA complexes in vitro, and that Rep likely provides this function in WT cells by forming a dual motor complex with the replicative helicase. The requirement for such activities demonstrates a critical need for nonrecombinogenic mechanisms to underpin replication fork movement in protein-rich environments and provides insight into the multiple ways in which such activities may be provided.

### Requirement for an Accessory Replicative Helicase In Vivo

We found that cells lacking both Rep and UvrD were inviable under rapid growth conditions but that this inviability was suppressed by growth on minimal medium (Figure 2A). Suppression was also achieved by mutations known to destabilize transcription complexes (Figures 2Ba and 3), reflecting a general hypersensitivity to the presence of high-affinity nucleoprotein complexes rather than any direct effects on transcription (Figure 4). Both Rep and UvrD also promoted resumption of replication by forks blocked at protein-DNA complexes in vitro (Figure 1), while possession of this in vitro activity correlated with the ability of helicases to complement Δ*rep* Δ*uvrD* lethality (Figure 5). Moreover, accumulation of toxic recombination intermediates in Δ*rep* Δ*uvrD* cells, although somewhat deleterious, was not the primary cause of inviability (Figures 2B and 2D), consistent with the ability of RuvABC, present in Δ*rep* Δ*uvrD* cells, to remove such intermediates in the absence of UvrD (Magner et al., 2007). We conclude that, under rapid growth conditions, fork movement along protein-bound template must be underpinned either by Rep or UvrD and that, in the absence of both these helicases, efficient chromosome duplication cannot be effected by the replicative helicase DnaB alone.

Might accessory replicative helicase activity be essential in all organisms? *S. cerevisiae* cells lacking the probable accessory replicative helicase Rrm3p are viable (Keil and McWilliams, 1993), arguing against such a requirement, but our data imply that other helicases might be able to substitute at least partially for Rrm3p in *rrm3* cells. Indeed, while Rrm3p is needed to minimize replisome pausing at many nonhistone protein-DNA complexes (Ivessa et al., 2003), it has little impact on pausing within highly transcribed RNAP II genes (Azvolinsky et al., 2009). Other helicases might therefore promote fork movement through such genes in the absence of Rrm3p.

### Action of Rep and UvrD at Replication Forks

Promotion of fork movement by accessory replicative helicases could conceivably occur by direct targeting of forks or by movement of helicases along duplex DNA remote from forks. However, neither Rep nor UvrD possessed the ability to displace EcoRI E111G from unreplicated DNA (Figure S2), consonant with both helicases being ssDNA translocases (Matson, 1986; Yarranton and Gefter, 1979). Thus, promotion of fork movement by Rep and UvrD occurred via targeting of blocked forks directly. Indeed, replication forks are the only plausible means of providing ssDNA for Rep/UvrD binding and subsequent translocation. The 3′-5′ polarity of Rep and UvrD and the critical contacts made with at least five nucleotides of ssDNA (Korolev et al., 1997; Lee and Yang, 2006) imply that direct targeting of forks requires a minimum of five nucleotides exposed on the leading strand template at the fork, ahead of the leading strand polymerase.

Polarity of helicase translocation also appears critical in promotion of fork movement. A third helicase with 3′-5′ polarity along ssDNA promoted fork movement in vitro and complemented Δ*rep* Δ*uvrD* lethality in vivo, whereas those with 5′-3′ polarity did neither (Figure 5). Thus, if 3′-5′ helicases translocate along the leading strand template to displace blocking nucleoprotein complexes, translocation along the lagging strand template by 5′-3′ helicases may be restricted. Such a restriction might be due to insufficient ssDNA being available on the lagging strand template for accessory helicase binding due to occlusion of this ssDNA by DnaB. This model implies that the template strand to which the replicative helicase binds might dictate the required polarity for ssDNA-specific helicases to act as accessory replicative helicases. Eukaryotic replicative helicases have a polarity opposite to that of bacterial DnaB (Forsburg, 2004). Helicases that promote fork movement might therefore require 5′-3′ translocation along ssDNA in eukaryotes, opposite to that determined for a bacterial system in this study. *S. cerevisiae* Rrm3p, the only eukaryotic helicase for which evidence exists of accessory replicative helicase function, is a 5′-3′ helicase that translocates along ssDNA, consistent with our proposal (Ivessa et al., 2002).

### Why Does the Replicative Helicase Require Backup?

Why might recruitment of Rep or UvrD succeed in promoting fork movement through a protein-DNA complex when DnaB translocation has failed? Successive loading of helicases, one behind the other, may promote protein displacement (Byrd and Raney, 2004). While there is no known mechanism to load additional DnaB hexamers onto a replisome to provide such assistance, probably as a consequence of the need to control replication initiation (Heller and Marians, 2006), binding of successive monomers of Rep or UvrD at a blocked fork could facilitate protein displacement. Tight control of replicative helicase loading is a conserved feature of replication systems, suggesting that similar considerations may apply to all organisms.

### Generalized and Fork-Specific Helicases Can Underpin Replication of Protein-Bound DNA

Rep and UvrD underpinned fork movement along protein-bound DNA in vitro (Figures 1B–1F), and each helicase provided this function in vivo in the absence of the other enzyme (Figures 2 and 4). However, low-level expression of Rep, but not UvrD, complemented Δ*rep* Δ*uvrD* lethality (Figures 5E and 7D), a property that correlated with the ability of Rep to interact physically and functionally with DnaB (Figures 6A–6C, 6E–6H, S4, and S5). Moreover, lack of this interaction abrogated Rep accessory helicase function in vitro and inhibited complementation of *Δrep ΔuvrD* lethality in vivo (Figure 7). These data indicate that Rep, present in low abundance in vivo (Scott and Kornberg, 1978), acts as the accessory replicative helicase in WT cells via physical and functional interaction with DnaB. Inhibition of the Rep-DnaB interaction by DnaC (Figures 6I and 6J) implies that Rep-DnaB complex formation likely occurs only after the initiation of replication. In contrast, UvrD may function as an accessory helicase only in the absence of Rep by virtue of the high abundance of UvrD (Janion, 2001). UvrD might therefore act as a generalized protein displacement motor during DNA replication in addition to nucleotide excision repair and recombination (Orren et al., 1992; Veaute et al., 2005). However, regardless of helicase priority, our data demonstrate that accessory replicative helicase function may be provided by both a fork-specific and a non-fork-specific motor.

### Replication Fork Progression in a Protein-Rich Environment

Displacement of blocking proteins out of the path of advancing replication forks by an accessory helicase obviates the need for recruitment of recombination enzymes and the attendant risks of genome rearrangements. Minimizing recombination may be critically important, given the frequency with which collisions must occur between replication forks and nucleoprotein complexes (Ivessa et al., 2003), especially transcribing RNAPs (Azvolinsky et al., 2009; Trautinger et al., 2005). In contrast, although recombination is important in facilitating replication of template containing DNA damage, excision repair mechanisms ensure that replisome collisions with DNA lesions are infrequent, except when such damage is extensive (Rupp et al., 1971). Thus, although recombination may be essential for survival in the face of programmed replication fork barriers located ectopically (Bidnenko et al., 2006; Lambert et al., 2005), accessory replicative helicases may act as a first line of defense against “accidental” fork blockage by nucleoprotein complexes. Our data therefore imply that such helicases may be ubiquitous, allowing genome duplication to occur concurrently with other essential DNA metabolic functions, and that within a single organism, multiple helicases may be able to perform such a function.

## Experimental Procedures

### Plasmids, Proteins, and Strains

*oriC*-containing plasmids bearing zero, two, or eight EcoRI sites (pPM436, pME101, and pPM594) and plasmids for the inducible expression of helicase genes (pBAD) were constructed as described in thw Supplemental Experimental Procedures. pAM403 is a pRC7 derivative encoding Rep (Mahdi et al., 2006). To generate pAM407, the promoter plus open reading frame of *uvrD* was amplified from MG1655 using primers incorporating ApaI sites and cloned into the ApaI site of pRC7.

Purification of proteins, surface plasmon resonance, pull-down, band-shift, and fork unwinding assays were performed as described in the Supplemental Experimental Procedures. Strains are listed in Table S1.

### In Vitro Replication Assays

Assays were performed in 40 mM HEPES (pH 8); 10 mM DTT; 10 mM magnesium acetate; 150 mM potassium glutamate; 2 mM ATP; 0.2 mM GTP, CTP, and UTP; 0.04 mM deoxyribonucleotides; and 0.1 mg ml^−1^ bovine serum albumin. Reactions (15 μl) contained 2 nM plasmid template, 50 nM DNA polymerase III αɛθ complex, 25 nM τ clamp loader complex, 160 nM DnaB and DnaC monomers, 1 μM SSB, 80 nM β, 30 nM HU, 200 nM DnaG, 300 nM DnaA, and the indicated concentrations of EcoRI E111G. Reactions were assembled on ice and initiated by addition of DnaA and incubation for 3 min at 37°C, followed by addition of 1 μl containing 47 units of EagI plus 0.4 MBq [α^32^P]dCTP (222 TBq/mmol). After a further 1.5 min at 37°C, unlabeled dCTP was then added to a final concentration of 4 mM. This unlabeled dCTP inhibited further incorporation of [α^32^P]dCTP. The test helicase was also added at this point, and incubation continued for 2 min at 37°C. Reactions were then terminated by addition of 1 μl of 0.5 M EDTA, and replication products were analyzed by denaturing agarose gel electrophoresis (Hiasa and Marians, 1994), phosphorimaging, and autoradiography. Efficiency of replication blockage (Figure S1) was analyzed in an identical manner, except that the indicated concentrations of E111G were employed, and after incubation with EagI and [α^32^P]dCTP for 1.5 min, reactions were terminated. 5′-labeled HindIII-digested λ DNA was used as a marker.

### Plasmid Loss Assays

Stock cultures of strains carrying derivatives of pRC7, maintained using 50 μg ml^−1^ ampicillin, were diluted 80-fold in LB broth and grown at 37°C with no ampicillin selection to A_650_ 0.4 before plating dilutions on LB agar or 56/2 glucose minimal agar supplemented with X-gal (120 μg ml^−1^) and IPTG (1 mM). Plates were photographed and scored after 48 hr (LB agar) or 72 hr (minimal agar) incubation at 37°C.

### Viability Assays and Identification of Suppressors

Plasmid free (*lac*^−^, Ap^s^) segregants of constructs carrying pRC7 derivatives were identified on 56/2 glucose minimal agar supplemented with X-gal and IPTG. These plasmid-free segregants were grown overnight in 56/2 glucose minimal salts medium. The next day, these cultures were diluted 20-fold in fresh salts medium and grown to an A_650_ of 0.4. Serial 10-fold dilutions of these cultures, from 10^−1^ to 10^−5^, were made with 56/2 salts, and then 10 μl aliquots were spotted onto LB agar plates and 56/2 glucose minimal agar plates. Plates were then incubated for 24 hr (LB agar) or 48 hr (minimal agar) at 37°C before photographing the plates. Spontaneously arising suppressors of the conditional inviability of *uvrD rep* strains were identified as rare healthy colonies arising on LB agar.

To analyze sensitivity to an array of repressor-operator complexes, pPM306 (Payne et al., 2006) was introduced into PM462–465 and colonies selected on LB agar containing 100 μg ml^−1^ ampicillin at 37°C overnight. Single colonies were then grown in LB broth plus ampicillin at 37°C to an A_650_ of 0.4. Serial dilutions were made as above before spotting 5 μl aliquots onto LB agar containing ampicillin and 0.2% arabinose ± 1 mM IPTG. Plates were photographed after 24 hr at 37°C.

Complementation of Δ*rep* Δ*uvrD* lethality using helicase genes was performed as described in the Supplemental Experimental Procedures.

## Supplemental Data

Document S1. Supplemental Experimental Procedures, Supplemental References, Six Figures, and One Table
