# Supplementary material for: Rep Provides a Second Motor at the Replisome to Promote Duplication of Protein-Bound DNA
Source: Mol Cell. 2009 Nov 25;36(4):654–66. doi: 10.1016/j.molcel.2009.11.009 (PMC2807033; doi:10.1016/j.molcel.2009.11.009)
Supplement: Document S1. Supplemental Experimental Procedures, Supplemental References, Six Figures, and One Table [file mmc1.pdf]

**Supplemental Data**
Molecular Cell, *Volume 36*

# Rep Provides a Second Motor at the Replisome to Promote Duplication of Protein-Bound DNA

Colin P. Guy, John Atkinson, Milind K. Gupta, Akeel A. Mahdi, Emma J. Gwynn, Christian J. Rudolph, Peter B. Moon, Ingeborg C. van Knippenberg, Chris J. Cadman, Mark S. Dillingham, Robert G. Lloyd, and Peter McGlynn

## Supplemental Experimental Procedures

### Plasmids

pPM436 was constructed by cleavage of pPM308 (Payne et al., 2006) with EcoRI, treatment with Klenow enzyme and deoxyribonucleotides followed by ligation to form an *oriC*-containing plasmid lacking EcoRI sites. pME101 was formed by cloning of a 288 bp PCR fragment flanked by EcoRI sites into the EcoRI site of pPM308. To generate pPM594, complementary oligonucleotides encoding 4 EcoRI sites were cloned into the XbaI site of pBluescript II SK(-) (Stratagene) to generate pPM528, then an EcoRI linker was cloned into the SmaI site of pPM528 to create pPM591. The XbaI-PvuII fragment of pPM591, encoding 6 EcoRI sites, was then filled in with Klenow and cloned into the Klenow-treated EcoRI site of pPM308 to form pPM594. Ligation of blunted XbaI and PvuII ends to blunted EcoRI sites generated two new EcoRI sites and so pPM594 contained a total of 8 EcoRI sites spanning a 280 bp region, confirmed by sequencing.

To generate plasmids for the inducible expression of helicase genes, firstly the kanamycin resistance gene from pKRP11 (Reece and Phillips, 1995) was cloned as a Klenow-treated HindIII fragment into the ScaI site of pBAD24 (Guzman et al., 1995) to create pBAD, conferring resistance to kanamycin but sensitivity to ampicillin. pBAD*rep* was formed by ligation of a Klenow-treated NdeI-BamHI fragment derived from pRH72, a pET21a clone encoding *rep* (Heller and Marians, 2005) into pBAD cleaved with XmaI and treated with Klenow. pBAD*repΔC33* was formed as follows: pMG19 (see below) and pBAD were cleaved with NdeI, treated with Klenow, and subsequently cleaved with PstI. The pMG19 *repΔC33*-containing fragment was then ligated into pBAD. pBAD*uvrD* was formed by ligation of a fragment, generated by cleavage of pETDuet*uvrD* (a kind gift of Nigel Savery, University of Bristol) with XhoI, treatment with Klenow and then cleavage with NcoI, into pBAD cleaved with NcoI and SmaI. pBAD*pcrA* was formed by ligation of a Klenow-treated NdeI-NotI fragment from a pET22b clone containing *B. stearothermophilus pcrA* (kindly supplied by Panos Soultanas, University of Nottingham) into Klenow-treated EcoRI and SmaI sites of pBAD. pBAD*dda* was formed by PCR amplification of *dda* from

bacteriophage T4 DNA (generously provided by Richard Bowater, University of East Anglia) with primers encoding NcoI and XbaI sites at the 5' and 3' ends of the gene, respectively, and cloned into pBAD cut with NcoI and XbaI. DNA sequencing revealed no errors within the cloned *dda*. pBAD*recD2* was generated by amplification of *Deinococcus radiodurans recD2* from a pET22b clone in which codon usage had been optimised for expression in *E. coli* (Saikrishnan et al., 2008). Amplification was performed with primers encoding NcoI and SmaI sites, the PCR product was cloned into pBAD cleaved with the same two restriction enzymes and the clone checked by DNA sequencing.

A *rep* overexpression clone encoding an N-terminal peptide tag for biotinylation by BirA was constructed by PCR amplification of *rep* from MG1655 and cloning into pET22b as a NdeI-XhoI fragment. Complementary oligonucleotides, encoding the biotinylation target MSGLNDIFEAQKIEWHEGGG, were then annealed and cloned into the NdeI site of pET22b*rep* to generate pET22b*biorep*. A *repΔ2B* expression clone was generated by PCR amplification of *repΔ2B* using pRepOΔ2B as template (Cheng et al., 2002) and primers bearing BamHI and NdeI sites at the 5' end and XhoI and PstI sites at the 3' end. This amplification product was cloned as a BamHI-PstI fragment into pUC19 cleaved with the same enzymes to yield pPM676 from which the EcoRI-XhoI *rep* fragment was cloned into pET22b*biorep* cleaved with EcoRI and XhoI to generate pET22b*biorepΔ2B*. To generate a *rep2B* expression clone, the *rep* sequence corresponding to G373-G543 was amplified using primers incorporating a start codon and NdeI site within the upstream primer and a stop codon and XhoI site within the downstream primer and cloned into pET22b to yield pET22b*rep2B*. Introduction of the biotin target was performed as described above for wild type *rep*. A plasmid to express biotinylated RepΔC33 was generated by use of oligonucleotides to amplify *rep* lacking the final 33 codons and bearing BamHI and NdeI sites at the 5' end and XhoI and PstI sites at the 3' end, using pRH72 (Heller and Marians, 2005) as a template. This PCR product was cloned as a BamHI-PstI fragment into pACT2 cleaved with BamHI and XhoI to yield pMG19 from which the EcoRI-XhoI *rep* fragment was cloned into pET22b*biorep* cleaved with the same enzymes to generate pET22b*biorepΔC33*.

A *uvrD* expression clone encoding a biotinylation target was generated in a similar manner to that for full length Rep to yield pET22b*biouvrD*.

**Proteins**

EcoRI E111G, Rep, UvrD, PriA, RecG, β, HU, DnaB, DnaC, DnaG, SSB, and DnaA were purified as described (Atkinson et al., 2009; King et al., 1989; Mahdi et al., 2003; Marians,

1995; Payne et al., 2006). TRCF, *B. stearothermophilus* DnaB and PcrA, bacteriophage T4 Dda and *Deinococcus radiodurans* RecD2 were kind gifts of Nigel Savery (University of Bristol), Panos Soultanas (University of Nottingham), Kevin Raney (University of Arkansas for Medical Sciences) and Dale Wigley (CRUK). N-terminally biotinylated $C33^{Rep}$, equivalent to the C-terminal 33 residues of Rep, was synthesised by Alta Biosciences, Birmingham, UK. EcoRI K62E (EcoRI HF) and EagI were purchased from New England Biolabs.

The nine individual subunits of DNA polymerase III were overexpressed and purified (at 4°C) as follows. The $\alpha$ and $\varepsilon$ subunit genes were cloned by PCR amplification into pET22b and pET24a, respectively, and co-overexpressed in BL21(DE3) in the presence of both ampicillin and kanamycin. Cells (8 L) were grown in F medium (Kim and McHenry, 1996) at 37°C to an $A_{650}$ of 0.3, the temperature shifted to 25°C and, once the $A_{650}$ had reached 0.5, IPTG was added to 1 mM and growth continued for a further 3 1/2 hours. Cells were harvested by centrifugation and resuspended in 50 mM Tris-HCl pH 7.5 and 10% sucrose to a final volume of 60 ml prior to storage at -80°C. Cells were subsequently thawed and then lysed by the addition of EDTA to 20 mM, Tris-HCl pH 8.4 to 50 mM, KCl to 150 mM, DTT to 10 mM, PMSF to 0.1 mM, 215 mg of protease inhibitor cocktail (Sigma) and lysozyme to 0.2 mg/ml. Cells were left on ice for 10 minutes then Brij 58 added to a final concentration of 0.1%. Incubation was continued on ice for a further 30 minutes followed by centrifugation at 100,000g at 4°C for one hour. The supernatant was recovered and Polymin P added dropwise to 0.075% at 4°C with stirring for one hour. Nucleic acids were precipitated by centrifugation in a Sorvall SS34 rotor at 20,000 rpm for 20 minutes at 4°C. Solid ammonium sulphate was then added to the recovered supernatant to 25% saturation, stirred on ice for 20 minutes and then centrifuged as for the Polymin P precipitation. Ammonium sulphate was then added to 50% saturation and proteins precipitated as above. This pellet was resuspended in 50 mM Tris-HCl pH 7.5, 1 mM EDTA, 1 mM DTT (buffer A) and 0.1 mM PMSF to a final volume of 110 ml. The $\alpha\varepsilon$ complex was purified using Q-Sepharose, Superdex 200, heparin-agarose, dsDNA-cellulose and Mono-Q columns using buffer A plus NaCl. Purified $\alpha\varepsilon$ was stored in 100 mM NaCl, 50 mM Tris-HCl pH 7.5, 1 mM EDTA, 1 mM DTT and 20% glycerol at -80°C.

The $\theta$ subunit gene, *holE*, was cloned by PCR amplification into pET22b and overexpressed in BL21(DE3). Cells (8 L) were grown at 37°C in LB medium with ampicillin until the $A_{650}$ reached 0.4. IPTG was then added to 1 mM and growth continued at 37°C for a further three hours prior to harvesting the cells by centrifugation and resuspension in Tris and sucrose as described above. Lysis and nucleic acid precipitation was as described

above, except that the final concentration of Polymin P was 0.4%. The supernatant from the Polymin P precipitation was applied to a Q-Sepharose column equilibrated in buffer B (20 mM Tris-HCl pH 7.5, 20% glycerol, 0.5 mM EDTA, 2 mM DTT) plus 50 mM NaCl. θ eluted in the flow-through and was then applied to a heparin-agarose column equilibrated in buffer B plus 50 mM NaCl. Again θ eluted in the flow-through and was applied to a S-Sepharose column equilibrated in buffer B plus 50 mM NaCl. θ was again present in the flow-through and was then loaded on to a phosphocellulose column equilibrated in buffer C (10 mM sodium phosphate pH 6.5, 10% glycerol, 0.5 mM EDTA, 2 mM DTT). θ was eluted with a linear gradient of 10-200 mM sodium phosphate pH 6.5 in buffer C (Studwell-Vaughan and O'Donnell, 1993). Peak fractions (approximately 100 mM sodium phosphate) were collected, ammonium sulphate added to 50% saturation and precipitated proteins harvested as above. The precipitate was resuspended in buffer B and passed through a Sephacryl S-200 gel filtration column equilibrated in buffer B plus 200 mM NaCl. Peak fractions were pooled and buffer B plus 80% glycerol was added to give a final glycerol concentration of 50% prior to storage at -80°C.

The γ and τ subunits are expressed from the same gene, *dnaX*, with γ being a truncated form of τ (Blinkova et al., 1993; Kodaira et al., 1983). γ was expressed by cloning the γ-only reading frame into pET22b. Overexpression was performed in BL21(DE3)/pLysS by growth of cells in 8 L of F medium with ampicillin and chloramphenicol at 37°C until the $A_{650}$ reached 0.6. IPTG was added to 1 mM and incubation continued at 37°C for a further three hours. Cells were harvested and resuspended in Tris and sucrose as above. Cells were lysed and treated with 0.075% Polymin P as described above, then proteins within the supernatant were precipitated by addition of ammonium sulphate to 40%. The precipitate was backwashed with 50 mM Tris-HCl pH 7.5, 1 mM EDTA, 10% glycerol, 5 mM DTT (buffer D) plus 50 mM NaCl and 0.1 mM PMSF with 35% then 30% ammonium sulphate. The final pellet was resuspended in buffer D and purified through Q-Sepharose, Sephacryl S200, heparin-agarose and DEAE-Sepharose in buffer D plus NaCl. γ bound to all but heparin-agarose. Fractions from the DEAE-Sepharose column were pooled and dialysed into buffer D plus 200 mM NaCl and 40% glycerol prior to storage at -80°C.

The τ subunit was overexpressed from the wild type *dnaX* gene cloned into pET22b. Overexpression was performed in BL21(DE3). Cells were grown in 8 L of F medium plus ampicillin at 37°C until an $A_{650}$ of 0.6 was reached. IPTG was added to 1 mM and growth continued at 37°C for three hours. Cells were harvested and resuspended as above. Cells were lysed and treated with 0.075% Polymin P as described above, then ammonium sulphate was added to 40% saturation. After centrifugation, the pellet was resuspended in

buffer A and then backwashed with 35% then 30% ammonium sulphate in buffer A. The final backwashed pellet was resuspended in buffer A plus 50 mM NaCl, 0.1 mM PMSF and 55 mg of protease inhibitor cocktail. τ was purified on S-Sepharose, heparin-agarose and Superdex 200 columns in buffer A plus NaCl. To remove traces of γ, pooled fractions from the gel filtration step were loaded onto a Mono-S HR 5/5 column equilibrated in buffer A plus 50 mM NaCl. τ was eluted with a 20 column volume gradient of 50 to 300 mM NaCl in buffer A and dialysed against 100 mM NaCl, 50 mM Tris-HCl pH 7.5, 1 mM EDTA, 1 mM DTT and 50% glycerol prior to storage at -80°C.

The χ and ψ subunit genes were cloned into pET24a and pET22b, respectively, and co-overexpressed in BL21(DE3) in the presence of ampicillin and kanamycin. Cells (8 L) were grown in LB medium at 37°C to an $A_{650}$ of 0.8 at which point IPTG was added to 1 mM and growth continued for a further three hours at 37°C. Cells were harvested and resuspended in Tris and sucrose as described above. Lysis was performed as above and nucleic acids were precipitated with 0.4% Polymin P. Proteins were precipitated by addition of solid ammonium sulphate to the supernatant to 45% saturation. The resultant pellet was resuspended in 50 mM Tris-HCl pH 7.8, 1 mM EDTA, 5 mM DTT and 20% glycerol (buffer E) and dialysed against buffer E plus 20 mM NaCl. The dialysate was loaded onto a Q-Sepharose column equilibrated in buffer E plus 20 mM NaCl and eluted with a 10 column volume linear gradient of 20 to 200 mM NaCl in buffer E. Peak fractions containing χ and ψ were pooled and dialysed in 50 mM Tris-HCl pH 7, 1 mM EDTA, 5 mM DTT and 20 mM NaCl (buffer F) prior to loading onto a S-Sepharose column equilibrated in buffer F plus 20 mM NaCl. Proteins were eluted with a 10 column volume gradient of 20 to 200 mM NaCl in buffer F. Peak fractions containing χ and ψ were pooled and proteins were precipitated by addition of solid ammonium sulphate to 45% saturation. The pellet was resuspended in buffer F and then passed through a Sephacryl S-200 gel filtration column equilibrated in buffer F plus 20 mM NaCl. Peak fractions were pooled and dialysed against buffer F plus 20 mM NaCl and 50% glycerol prior to storage at -80°C.

The δ subunit gene was cloned into pET22b and overexpressed in BL21(DE3). Cells (8 L) were grown in LB with ampicillin at 37°C to an $A_{650}$ of 0.5. IPTG was added to 1 mM and growth continued for a further three hours at 37°C before harvesting and storage of cells as described above. Cells were then thawed, lysed, and nucleic acids precipitated with 0.4% Polymin P as described above. Proteins were precipitated with 50% ammonium sulphate and resuspended in 50 mM Tris-HCl pH 8, 1 mM EDTA and 20% glycerol (buffer G) to an equivalent conductivity of buffer G plus 50 mM NaCl. δ was then purified on Q-Sepharose, heparin-agarose and Sephacryl S-200 columns in buffer G plus NaCl prior to

storage in 50 mM Tris-HCl pH 7.5, 75 mM NaCl, 1 mM EDTA, 5 mM DTT and 50% glycerol.

The δ' subunit gene was cloned into pET22b and overexpressed as described for δ. Lysis, Polymin P and ammonium sulphate precipitation was also as for δ. Protein pellets after ammonium sulphate precipitation were resuspended in 50 mM Tris-HCl pH 8.5, 1 mM EDTA and 5 mM DTT (buffer H) and then dialysed against buffer H. δ' was purified by chromatography through Q-Sepharose, Sephacryl S-200 and heparin-agarose in buffer H plus NaCl prior to storage in 50 mM Tris-HCl pH 8, 50 mM NaCl, 1 mM EDTA, 2.5 mM DTT and 50% glycerol.

Biotinylated Rep was overexpressed by transformation of pET22b*biorep* into *E. coli* BL21(DE3) harbouring pBirAcm (Avidity, LLC), encoding a biotin ligase. Colonies containing both the pET22b and pBirAcm plasmids were selected using ampicillin (100 μg $ml^{-1}$) and chloramphenicol (25 μg $ml^{-1}$). A single colony was inoculated into 10 ml of LB broth containing ampicillin and chloramphenicol and grown for 8 hours at 37°C with shaking. Cells were harvested by centrifugation, resuspended in 10 ml of LB and 1 ml inoculated into 80 ml of LB containing ampicillin and chloramphenicol. After growth at 37°C with shaking overnight, the cells were pelleted, resuspended in fresh LB and inoculated into 8 l of LB in a stirred vessel fermenter containing the above antibiotics and grown at 37°C to an $A_{650}$ of 0.4. The culture was allowed to cool to 25°C and then IPTG and biotin were added to final concentrations of 1 mM and 500 μM, respectively, and incubation continued at 25°C for a further 3 hours. Cells were harvested by centrifugation and the cell pellet resuspended in 50 mM Tris-HCl pH 7.5 and 10% sucrose prior to storage at -80°C. Cells equivalent to 2 l of the culture were subsequently thawed and then lysed by the addition of EDTA to 1 mM, Tris-HCl pH 8.4 to 50 mM, KCl to 150 mM, DTT to 1 mM, PMSF to 1 mM and lysozyme to 0.2 mg/ml. Cells were left on ice for 10 minutes then Brij 58 added to a final concentration of 0.1%. Incubation was continued on ice for a further 30 minutes followed by centrifugation at 100,000g at 4°C for one hour. The supernatant was recovered and solid ammonium sulphate was then added to the recovered supernatant to 50% saturation, stirred on ice for 20 minutes and then centrifuged in a Sorvall SS34 rotor at 20,000 rpm for 20 minutes at 4°C. Precipitated protein was resuspended in buffer A (see above) plus 0.1 mM PMSF until a conductance equivalent to buffer A plus 100 mM NaCl was reached. The resuspension was loaded onto a 5 ml Softlink Avidin column (Promega) equilibrated in buffer A plus 100 mM NaCl. The column was washed first with buffer A plus 100 mM NaCl, then buffer A plus 1 M NaCl and finally buffer A plus 100 mM NaCl. Biotinylated protein was then eluted by flushing the column with buffer A containing

100 mM NaCl and 5 mM biotin, pausing the elution for 30 minutes just as protein began to appear in the eluate and then continuing the elution with the same buffer. Fractions containing biotinylated Rep were pooled and loaded onto a 1 ml Hi-trap heparin column (GE Healthcare) equilibrated in buffer A plus 100 mM NaCl, the column was washed with buffer A plus 100 mM NaCl and then protein eluted using a 100-1000 mM NaCl gradient. Positive fractions were then pooled and dialysed into 100 mM NaCl, 50 mM Tris-HCl pH 7.5, 1 mM EDTA, 1 mM DTT and 50% glycerol prior to storage at -80°C.

Biotinylated RepΔ2B, Rep2B, RepΔC33 and UvrD were overexpressed and purified in a similar manner except that all 8 l of the RepΔ2B culture was used for purification. RepΔ2B was also purified in a similar manner except that chromatography on heparin was replaced by Mono-Q (GE Healthcare).

**Reconstitution of Core Polymerase and Clamp Loader Complexes**

Core polymerase was reconstituted by mixing 1 mg of αε with 0.4 mg of θ in 20 mM Tris-HCl pH 7.5, 2 mM DTT, 0.5 mM EDTA and 20% glycerol (buffer I) plus 30 mM NaCl. Proteins were incubated at 4°C with gentle agitation for one hour. The mixture was then loaded onto a Mono-Q HR 5/5 column equilibrated in buffer I and αεθ eluted with a 40 column volume linear gradient of 0-400 mM NaCl (Onrust et al., 1995) in buffer I. Fractions were pooled and stored at -80°C in 20 mM Tris-HCl pH 7.5, 1 mM EDTA, 2 mM DTT, 180 mM NaCl and 40% glycerol.

τ clamp loader was reconstituted by mixing 1.1 mg of τ, 0.88 mg of δ, 0.87 mg of δ', and 1.1 mg of χψ in buffer I and incubating at 4°C with gentle agitation for one hour. The mixture was then purified on a Mono-Q HR 5/5 column as for core polymerase except that 80 column volumes were used for elution. Fractions containing all five proteins were pooled and stored in 50 mM Tris-HCl pH 7.5, 1 mM DTT, 1 mM EDTA, 100 mM NaCl and 50% glycerol at -80°C.

γ clamp loader was reconstituted as for the τ complex except that the τ subunit was replaced by 1.55 mg of γ.

**EcoRI Cleavage Assay**

To establish whether Rep or UvrD could displace E111G from unreplicated DNA, linearised pME101 was generated by cleavage with EagI followed by purification using a Qiagen spin column. Cleavage reactions were then performed on 200 ng of linearised DNA in 40 mM HEPES (pH 8), 10 mM DTT, 10 mM magnesium acetate, 150 mM

potassium glutamate, 2 mM ATP, 0.2 mM GTP, CTP and UTP, 0.04 mM deoxyribonucleotides and 0.1 mg $ml^{-1}$ bovine serum albumin in a total final volume of 10 µl. Reactions were preincubated for 2 minutes at 37°C prior to addition of EcoRI E111G, Rep and/or UvrD. Where E111G was added together with Rep or UvrD, proteins were premixed prior to addition to the reaction. Incubation was continued for 1 minute at 37°C. EcoRI K62E ("EcoRI-HF" from New England Biolabs Inc.) without and with E111G was then added and incubation continued for 1 minute at 37°C. In the reaction containing both restriction enzymes (Figure S1B, lane 9), the proteins were premixed prior to addition. Reactions were stopped by addition of 2.5 µl of 100 mM Tris.HCl pH 7.5, 200 mM EDTA and 10 mg/ml proteinase K. 2.5 µl of 30% glycerol plus 0.25% bromophenol blue was then added and reactions separated on 0.8 % agarose/TBE gels. DNA was visualised by staining with ethidium bromide. Final concentrations of proteins were 200 nM dimers of EcoRI E111G, 100 nM of Rep and UvrD and 1 unit/µl of EcoRI K62.

**Complementation of Δ*rep* Δ*uvrD* Lethality Using Helicase Genes**

pBAD plasmids encoding helicase genes were introduced into N6524 and N6556 and colonies bearing both pAM403 (pRC7*rep*) and pBAD plasmids were selected on LB agar containing ampicillin (50 μg $ml^{-1}$) and kanamycin (30 μg $ml^{-1}$) at 37°C overnight. Single colonies were restreaked onto 56/2 glucose minimal agar containing kanamycin, Xgal and IPTG. After 72 hours at 37°C all strains gave a mixture of blue and white colonies. Single white colonies were inoculated into 56/2 glucose minimal salts medium with kanamycin at 37°C to an $A_{650}$ of 0.6. Serial dilutions were made in 56/2 salts, 5 μl aliquots spotted onto LB agar containing kanamycin ± 0.2% arabinose and plates photographed after 24 hours at 37°C.

**Surface Plasmon Resonance**

Surface plasmon resonance was performed at 25°C on a BIAcore 2000 instrument. Immobilisation of *E. coli* and *B. stearothermophilus* DnaB was performed using EDC/NHS coupling onto CM5 chips at pH 4.0 whilst the indicated concentrations of Rep and UvrD were passed over DnaB in 40 mM HEPES (pH 8), 10 mM DTT, 10 mM magnesium acetate, 150 mM potassium glutamate and 0.1 mg $ml^{-1}$ bovine serum albumin at 20 μl $min^{-1}$. This buffer was identical to that used in the *in vitro* DNA replication assays except for the omission of nucleotides. This omission was required since the presence of nucleotides in the buffer generated random spikes in the resonance signal. Surface plasmon resonance with immobilised biotinylated Rep, mutant Rep proteins and $C33^{Rep}$ peptide was performed

by immobilisation onto streptavidin-coated SA sensor chips. Interaction of *E. coli* DnaB and DnaC with immobilised Rep proteins was analysed in 10 mM HEPES pH 7.4, 3 mM EDTA, 150 mM NaCl, 10 mM $MgCl_2$ and 0.005% Tween 20 at 20 μl $min^{-1}$. This buffer differed from that used in the *in vitro* DNA replication assays so as to minimise non-specific interactions with the surface-immobilised streptavidin.

**Pulldown Assays**

Cell lysate was prepared by growth of *E. coli* MG1655 in 1 l of LB at 37°C with shaking to an $A_{600}$ of 1.2, harvesting of cells by centrifugation and resuspension in 20 ml of 10% sucrose, 20 mM Tris-HCl pH 7.5, 1 mM EDTA, 1 mM DTT and 100 mM NaCl. Cells were lysed by sonication followed by addition of lysozyme to 0.5 mg $ml^{-1}$ and Triton X-100 to 0.1% and subsequent agitation at room temperature for 20 minutes. The lysate was then clarified by centrifugation.

Pulldown experiments were performed using streptavidin-coated magnetic beads (New England Biolabs). Each step was performed at 4°C by placing tubes on a rotator for the specified time prior to removal of wash solution by placing the tubes on a magnetic stand for two minutes. Initially 100 μl of the bead stock (4 mg $ml^{-1}$) were washed with 200 μl of pulldown buffer (20 mM Tris-HCl pH 7.5, 150 mM NaCl, 1 mM EDTA and 0.1% Triton X-100) for 5 minutes, then with 200 μl of biotinylated bait protein diluted to 1 μM in pulldown buffer containing 0.1% bovine serum albumin for 20 minutes. Beads were then washed with 200 μl of pulldown buffer plus 0.1% bovine serum albumin for 5 minutes before mixing with 200 μl of cell lysate. Incubation was continued for 20 minutes prior to removal of the cell extract, the beads were washed with 200 μl of pulldown buffer plus 0.1% bovine serum albumin for five minutes and then with 200 μl of pulldown buffer, again for 5 minutes. The beads were then boiled for 5 minutes in 50 μl of 62.5 mM Tris-HCl pH 6.8, 10% SDS, 25% glycerol, 0.7 M β-mercaptoethanol and 0.5% bromophenol blue, the beads removed and then 30 μl of the supernatant analysed by electrophoresis through 10% SDS polyacrylamide gels prior to staining with Coomassie Brilliant Blue R-250. Western blots were performed on gels in which 5 rather than 30 μl of supernatant was electrophoresed using rabbit antisera raised against purified DnaB together with HRP-conjugated mouse anti-rabbit antibodies and ECLPlus reagents (GE Healthcare). Coomassie-stained gels also contained 100 ng purified DnaB whilst gels for Western blotting contained 40 ng purified DnaB.

**Bandshift Assays**

A radioactively labelled forked DNA substrate having a 60 bp duplex and two 38 base ssDNA arms was formed from oligonucleotides 5 and 6 as described in (Atkinson et al., 2009). Bandshifts were performed in 50 mM HEPES pH 8, 10 mM magnesium acetate, 10 mM DTT, 10 μM ADP and 50 μg $ml^{-1}$ bovine serum albumin. Reactions containing 1 nM DNA substrate were preincubated for 2 minutes at 37°C prior to addition of proteins at the indicated concentrations to give a final volume of 10 μl. Incubation was continued for 10 minutes at 37°C prior to addition of 2 μl of 30% glycerol. Reactions were then loaded onto a 4% polyacrylamide gel with 89 mM Tris base, 89 mM boric acid and 10 μM ADP as running buffer this and then electrophoresis performed at 160 V for 90 minutes at room temperature prior to drying and analysis by autoradiography and phosphorimaging.

**Fork Unwinding Assays**

The forked DNA substrate to analyse cooperativity between Rep and DnaB was that used for the bandshift assays described above. Reactions contained 1 nM DNA substrate in 50 mM HEPES (pH 8), 10 mM DTT, 10 mM magnesium acetate, 10 mM DTT, 2 mM ATP and 0.2 mg $ml^{-1}$ bovine serum albumin and were assembled on ice in the absence of proteins, then incubated at 37°C for 2 minutes. DnaB was then added as indicated and incubation continued for 2 minutes at 37°C prior to addition of Rep and UvrD as indicated to give a final reaction volume of 10 µl. Incubation was continued for 10 minutes at 37°C then reactions terminated by addition of 2.5 µl of 100 mM Tris.HCl pH 7.5, 200 mM EDTA, 10 mg/ml proteinase K and 0.5% SDS. 2.5 µl of 30% glycerol plus 0.25% bromophenol blue was then added and reactions separated on 10% polyacrylamide/TBE gels prior to analysis of dried gels by phosphorimaging and autoradiography. Cooperativity of unwinding was analysed by dividing the fraction of the DNA substrate unwound by Rep (or UvrD) in the presence of DnaB by the sum of the fractions of DNA unwound by Rep (or UvrD) alone and by DnaB alone. Values approximating 1 indicate no enhancement (or inhibition) of unwinding upon incubation with both Rep (or UvrD) and DnaB. Values above 1 indicate enhancement of unwinding upon co-incubation of the DNA substrate with the two indicated helicases. Comparison of Rep versus RepΔC33 helicase activity was performed in a similar manner except that the forked DNA substrate was formed from oligonucleotides 1 and 2 described in (Cadman et al., 2006).

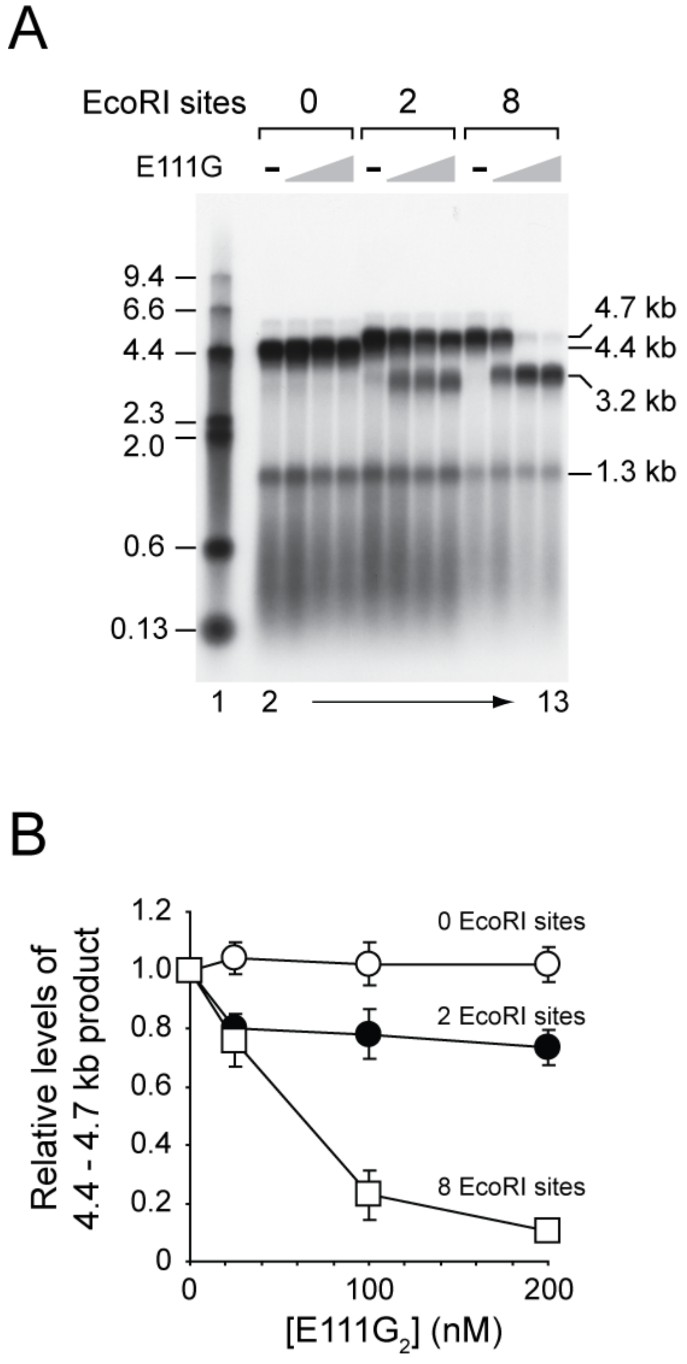


Supplementary Figure 1

**Figure S1. Blockage of Replication Forks In Vitro by EcoRI E111G-DNA Complexes**

(A) Denaturing agarose gel of replication products formed with *oriC*-containing plasmids bearing 0, 2 or 8 EcoRI sites performed as shown in Figure 1A except that reactions were terminated prior to addition of unlabelled dCTP and candidate helicases. Reactions contained 0, 25, 100 or 200 nM E111G dimers, as indicated. Sizes of HindIII-cut λDNA, in kb, are noted on the left and estimated sizes of leading strand products on the right. Note that whilst the full length leading strands generated from the plasmids bearing 2 and 8 EcoRI sites (pME101 and pPM594 respectively) were 4.7 kb, the full length leading strand generated from pPM436 containing no EcoRI sites was 4.4 kb.

(B) Relative levels of the 4.4-4.7 kb leading strand as a function of E111G concentration relative to control reactions in lanes 2, 6 and 10 in (A). Error bars represent standard deviation of the mean.

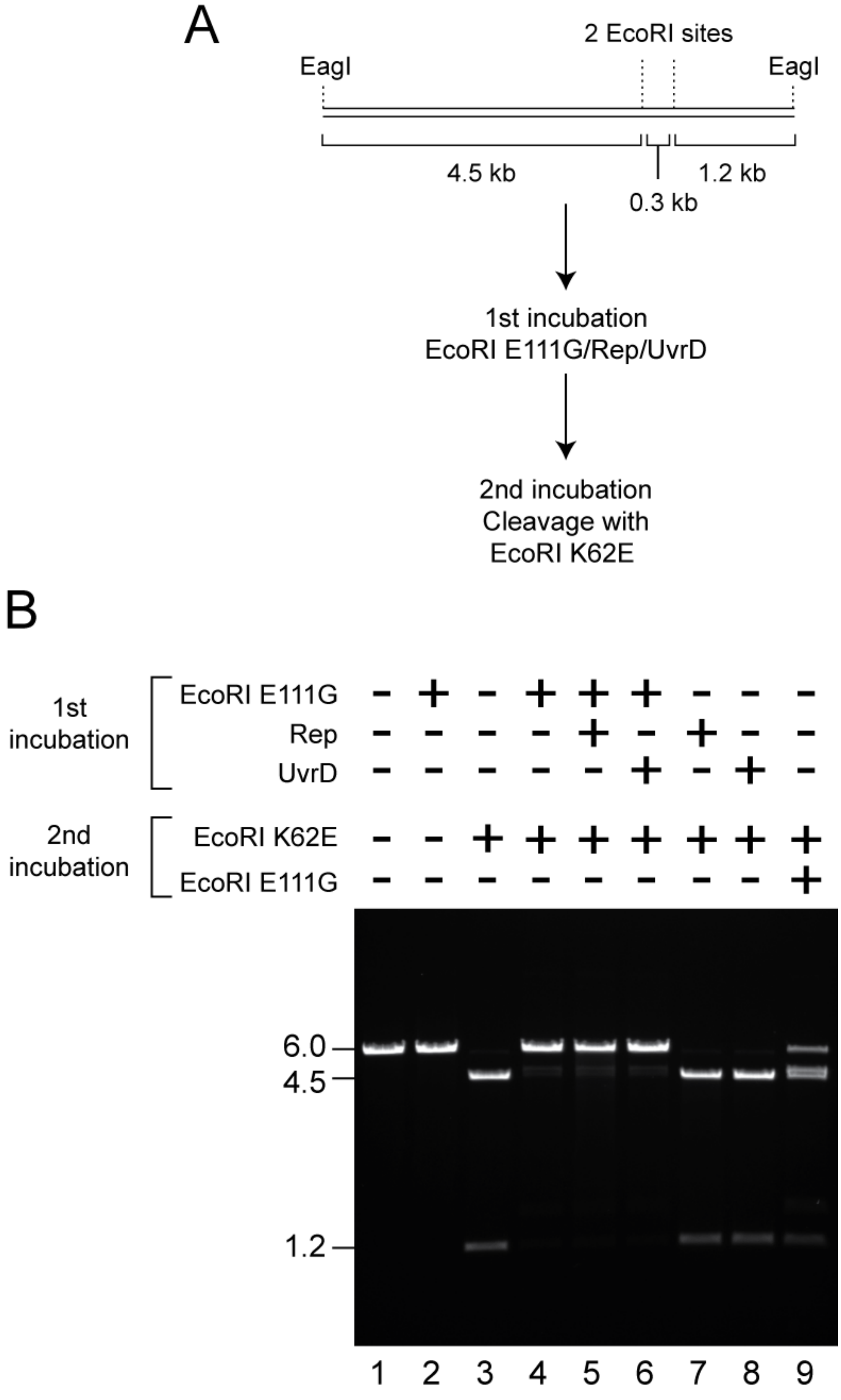


Supplementary Figure 2

**Figure S2. Rep and UvrD Cannot Displace EcoRI E111G from Unreplicated DNA**

(A) pME101, containing 2 EcoRI sites and as used in Figure 1D, was linearised by cleavage with EagI as for the *in vitro* replication reactions and processed as indicated.

(B) Ethidium bromide-stained agarose gel of EcoRI K62E cleavage products obtained from the linearised duplex. The purified linearised duplex was incubated with E111G, Rep and/or UvrD for 1 minute at 37°C. EcoRI K62E with and without E111G was then added and incubation continued at 37°C for a further 1 minute prior to deproteinisation of the reactions and electrophoresis. EcoRI K62E (New England Biolabs Inc.) has the same cleavage specificity as wild type EcoRI but has reduced star activity, with cleavage of the linear duplexes generating the expected 4.5 and 1.2 kb products (lane 3). Note that the 0.3 kb product was not resolved under these conditions. No cleavage of the template was observed with E111G (compare lanes 1 and 2), as expected (King et al., 1989). Preincubation of the DNA with E111G prior to addition of EcoRI K62E resulted in significant inhibition of cleavage (compare lanes 3 and 4) demonstrating that binding of E111G to the EcoRI sites inhibited access by EcoRI K62E. Addition of Rep or UvrD simultaneously with E111G provided no detectable relief of this inhibition (lanes 5 and 6). This lack of relief was not due to direct inhibition of EcoRI K62E cleavage by Rep or UvrD as neither helicase inhibited cleavage in the absence of E111G (lanes 7 and 8). Furthermore, high levels of cleavage were still obtained upon simultaneous addition of E111G and EcoRI K62E, although cleavage was reduced compared to those obtained in the absence of E111G (compare lanes 3 and 9). EcoRI K62E could therefore compete with E111G in the absence of prior binding of E111G. The inability of either Rep or UvrD to promote cleavage indicates therefore that neither helicase can promote efficient displacement of E111G from unreplicated DNA.

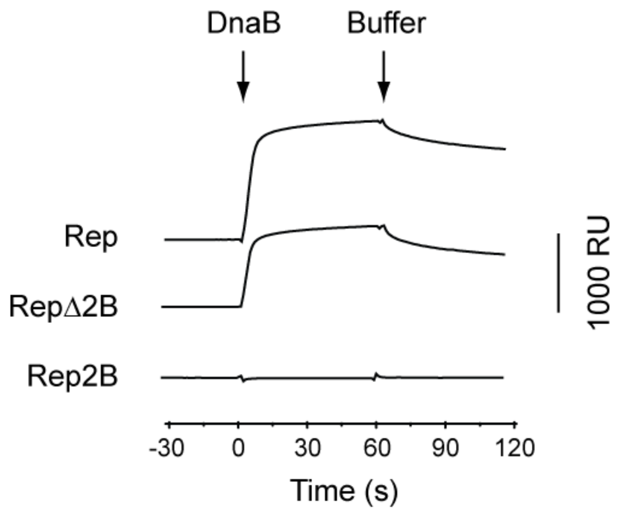


Supplementary Figure 3

**Figure S3**.

Interaction of surface immobilised biotinylated Rep, a mutant Rep protein lacking the 2B subdomain (RepΔ2B) and the isolated Rep 2B subdomain (Rep2B) with *E. coli* DnaB. 8100, 7600 and 7100 resonance units of Rep, RepΔ2B and Rep2B were surface immobilised onto streptavidin-coated chips. DnaB was present at 4 μM monomers.The Rep2B subdomain provides an autoregulatory mechanism to control DNA unwinding, possibly mediated via unidentified protein-protein interactions (Brendza et al., 2005). However, RepΔ2B retained the ability to interact with DnaB whilst there was no detectable interaction between the isolated 2B subdomain and DnaB. Interaction between DnaB and Rep was not therefore mediated via the Rep 2B subdomain.

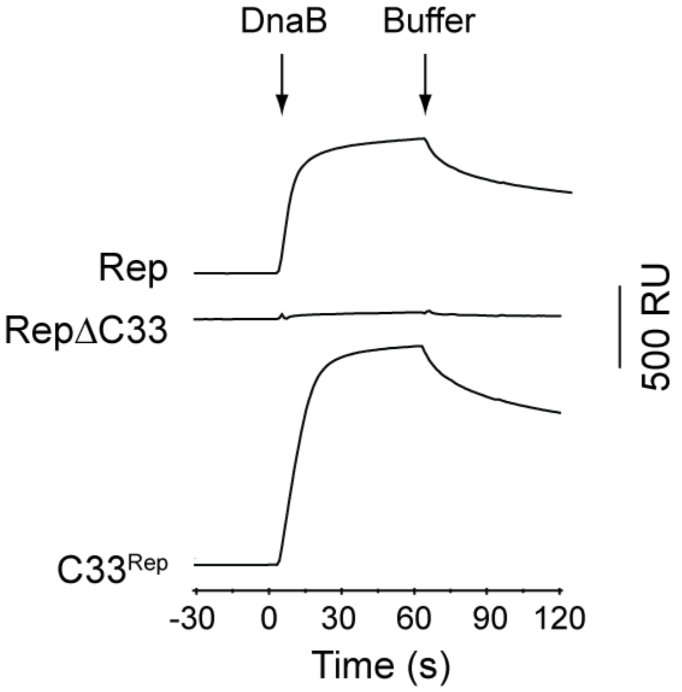


Supplementary Figure 4

**Figure S4**.

Interaction of *E. coli* DnaB with surface-immobilised wild type Rep, RepΔC33 and a synthetic peptide corresponding to the 33 C-terminal residues of Rep ($C33^{Rep}$). 4000 resonance units of Rep and RepΔC33 and 280 resonance units of $C33^{Rep}$ were immobilised whilst DnaB was present at 1 μM (monomers).

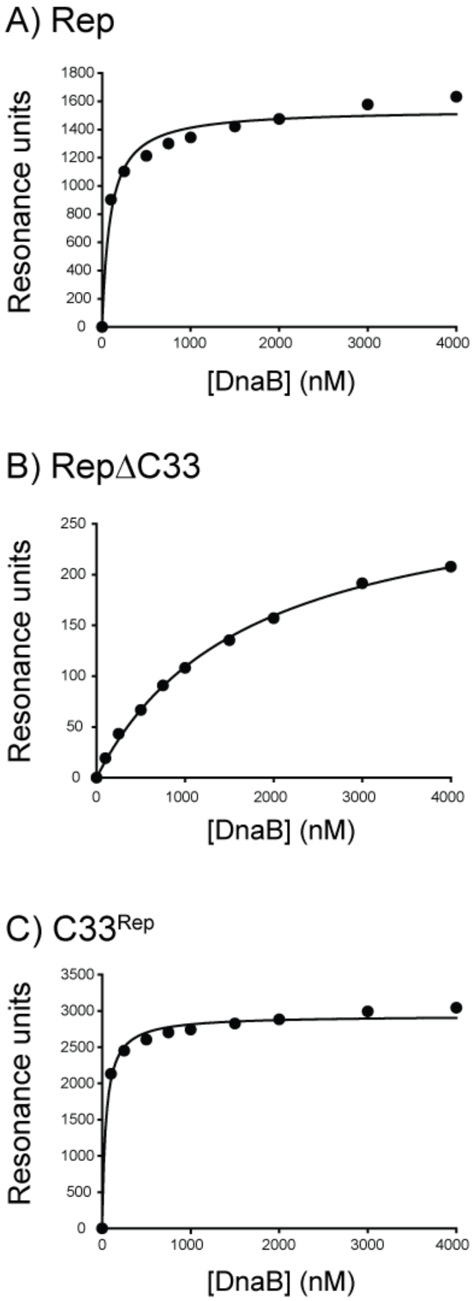


Supplementary Figure 5

**Figure S5. Affinity of DnaB for Rep, RepΔC33, and C33$^{Rep}$**

The indicated monomer concentrations of DnaB were analysed by SPR against Rep, RepΔC33 and C33$^{Rep}$ immobilised via biotinylated N-termini as described in Experimental Procedures. The fit for a 1:1 binding model is shown with $R^2$ values of 0.97, 1.00 and 0.99 obtained for Rep, RepΔC33 and C33$^{Rep}$ respectively. Estimated apparent $K_D$ (equilibrium dissociation constant) values of 93, 44 and 1712 nM were obtained for Rep, C33$^{Rep}$ and RepΔC33, respectively. Note that data using concentrations of DnaB less than 100 nM could not be obtained. The number of resonance units obtained below 100 nM DnaB approached background levels possibly due to inefficient DnaB hexamer formation at these concentrations. Data were collected using 4084, 3910 and 214 resonance units of immobilised Rep, RepΔC33 and C33$^{Rep}$ respectively whilst DnaB was passed over at 20 μl $min^{-1}$ for 400 s to obtain steady-state resonance values.

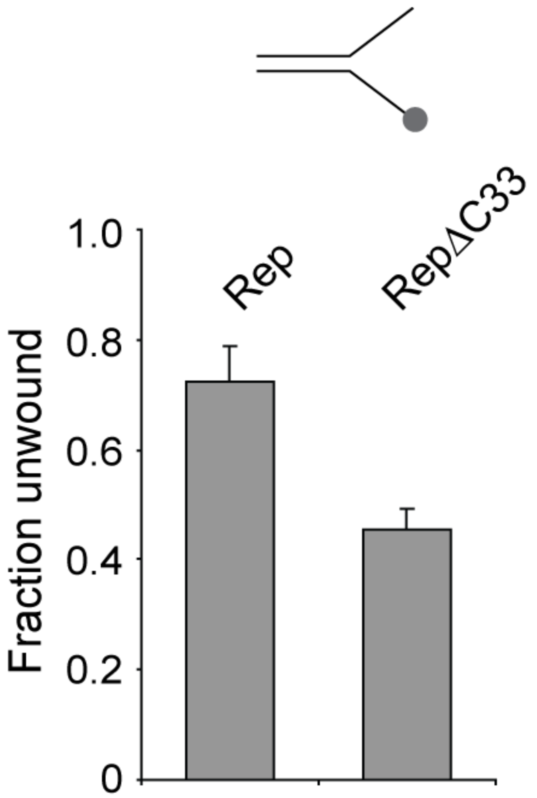


Supplementary Figure 6

**Figure S6**

Helicase activity of Rep versus RepΔC33 (both at a concentration of 50 nM and bearing an N-terminal biotin tag) as measured by unwinding of a forked DNA substrate having a 25 bp duplex and 25 base ssDNA arms. Error bars represent standard deviation of the mean.

## Table S1. *Escherichia coli* K-12 Strains

| Strain | Relevant Genotype | Source |
|---|---|---|
| **(a) General P1 donors** | | |
| AB1157 | *araC14 thi-1 hisG4 Δ(gpt-proA)62 argE3 thr-1 leuB6 kdg51 rfbD1 lacY1 galK2 xyl-5 mtl-1 tsx-33 supE44 rac*$^-$ *mgl-51 rpsL31 qsr*$^-$ | (Bachmann, 1996) |
| CGSC4518 | *pyrE60 argE3 his-4 proA2 thr-1 leuB6 mtl-1 xyl-5 araC14 galK2 lacY1 str-31 supE44* | *E. coli* Genetic Stock Centre |
| JC12334 | *tnaA300*::Tn*10 recF143* | A. J. Clark |
| JJC735 | *Δrep*::*cat* | Bénédicte Michel |
| KL14 | Hfr (PO68) *thi-1 relA1 spoT1* | (Bachmann, 1996) |
| LMG194 | KS272 *Δara714 leuB::*Tn*10* | (Guzman et al., 1995) |
| N3072 | *recA269*::Tn*10* | (Lloyd et al., 1987) |
| N3793 | *ΔrecG263*::*kan* | (Al-Deib et al., 1996) |
| N4147 | *araC14 thi-1 hisG4 Δ(gpt-proA)62 thr-1 leuB6 kdg51 rfbD1 lacY1 galK2 xyl-5 mtl-1 tsx-33 supE44 rac*$^-$ *mgl-51 rpsL31 qsr*$^-$ *spoT1* | AB1157 x Hfr KL14 to Arg$^+$ (Str$^r$) |
| N4339 | *pyrE60 argE3 his-4 proA2 thr-1 leuB6 mtl-1 xyl-5 araC14 galK2 lacY1 str-31 supE44 ΔrecG263*::*kan* | P1.N3793 x CGSC4518 to Km$^r$ |
| **(b) MG1655 and derivatives**[a] | | |
| MG1655 | F$^-$ *rph-1* | (Bachmann, 1996) |
| AM1657 | *ΔuvrD*::*dhfr* | This work[b] |
| AM1742 | pAM407 *(lac*$^+$ *uvrD*$^+$*) / ΔlacIZYA* | This work |
| AM2073 | *ΔlacIZYA ΔrecG*::*apra zjf-920*::Tn*10 ΔpriB202* $rpoB_{G3779A}$ | AAM and RGL unpublished work |
| AM2155 | *ΔlacIZYA argE86*::Tn*10* | P1.N4837 x TB28 to Tc$^r$ |
| AM2156 | *ΔlacIZYA argE86*::Tn*10* $rpoB_{A1714T}$ (Rif$^r$) | Selection of AM2155 for high level resistance to rifampicin |
| AM2158 | *ΔlacIZYA* $rpoB_{G3779A}$ | P1.AM2073 x AM2156 to Arg$^+$ and Rif$^s$ |
| BP41 | *Δara714 argEC::[apra*$^r$ $lacO_{34}$*]* | (Payne et al., 2006) |
| N4837 | *argE86*::Tn*10* | (Jaktaji and Lloyd, 2003) |
| N4934 | *recJ284*::Tn*10* | (Mahdi et al., 2006) |
| N5014 | *pyrE60 ΔrecG263*::*kan* | P1.N4339 x MG1655 to Km$^r$ and uracil-requiring (*pyrE60*) |
| N5020 | *spoT1* | P1.N4147 x N5014 to Pyr$^+$ (Km$^s$) |
| N5584 | *Δrep*::*kan* | (Mahdi et al., 2006) |
| N5916 | *rpoB*35 ΔlacIZYA<>aph* | P1.TB12 x N4849 |

| | | |
|---|---|---|
| N5923 | *rpoB*35 ΔlacIZYA<>aph pCP20* | N5916 x pCP20 (Cherepanov and Wackernagel, 1995) to Ap$^{r}$ at 30°C |
| N5925 | *rpoB*35 ΔlacIZYA* | Plasmid free Km$^{s}$ segregant of N5923 identified after growth in LB broth at 42°C. |
| N5942 | pAM374 *(lac*$^{+}$ *priA*$^{+}$*)* / *priA300 ΔlacIZYA ΔrecQ*::*kan* | (Mahdi et al., 2006) |
| N6168 | pAM374 *(lac*$^{+}$ *priA*$^{+}$*)* / *priA300 ΔlacIZYA ΔrecQ*::*kan ΔuvrD*::*dhfr* | P1.AM1657 x N5942 to Tm$^{r}$ |
| N6499 | *priA300 ΔlacIZYA ΔrecQ*::*kan ΔuvrD*::*dhfr* | Plasmid free segregant of N6168 |
| N6524 | pAM403 *(lac*$^{+}$*rep*$^{+}$*)* / *ΔlacIZYA* | TB28 x pAM403 to Ap$^{r}$ |
| N6539 | pAM403 *(lac*$^{+}$*rep*$^{+}$*)* / *ΔlacIZYA Δrep*::*kan* | P1.N5584 x N6524 to Km$^{r}$ |
| N6540 | pAM403 *(lac*$^{+}$*rep*$^{+}$*)* / *ΔlacIZYA Δrep*::*cat* | P1. JJC735 x N6524 to Cm$^{r}$ |
| N6552 | pAM403 *(lac*$^{+}$*rep*$^{+}$*)* / *ΔlacIZYA Δrep*::*cat ΔrecQ*::*kan ΔuvrD*::*dhfr* | P1.N6499 x N6540 to Km$^{r}$ (Tm$^{r}$) |
| N6556 | pAM403 *(lac*$^{+}$*rep*$^{+}$*)* / *ΔlacIZYA Δrep*::*cat ΔuvrD*::*dhfr* | P1.AM1657 x N6540 to Tm$^{r}$ |
| N6568 | pAM403 *(lac*$^{+}$*rep*$^{+}$*)* / *ΔlacIZYA ΔuvrD*::*dhfr* | P1.AM1657 x N6539 to Tm$^{r}$ (Km$^{s}$ *rep*$^{+}$) |
| N6577 | *ΔlacIZYA Δrep*::*cat* | Plasmid free segregant of N6540 |
| N6632 | *ΔlacIZYA ΔuvrD*::*dhfr* | Plasmid free segregant of N6568 |
| N6639 | pAM407 *(lac*$^{+}$ *uvrD*$^{+}$*)* / *ΔlacIZYA ΔuvrD*::*dhfr* | P1.AM1657 x AM1742 to Tm$^{r}$ |
| N6643 | pAM407 *(lac*$^{+}$ *uvrD*$^{+}$*)* / *ΔlacIZYA ΔuvrD*::*dhfr Δrep*::*kan* | P1.N5584 x N6639 to Km$^{r}$ |
| N6644 | pAM407 *(lac*$^{+}$ *uvrD*$^{+}$*)* / *ΔlacIZYA ΔuvrD*::*dhfr Δrep*::*cat* | P1. JJC735 x N6639 to Cm$^{r}$ |
| N6645 | pAM407 *(lac*$^{+}$ *uvrD*$^{+}$*)* / *ΔlacIZYA ΔuvrD*::*dhfr ΔpriC303*::*kan* | P1.JJC1405 x N6639 to Km$^{r}$ |
| N6661 | pAM407 *(lac*$^{+}$ *uvrD*$^{+}$*)* / *ΔlacIZYA Δrep*::*kan* | P1.N5584 x AM1742 to Km$^{r}$ |
| N6662 | pAM407 *(lac*$^{+}$ *uvrD*$^{+}$*)* / *ΔlacIZYA Δrep*::*cat* | P1. JJC735 x AM1742 to Cm$^{r}$ |
| N6669 | pAM407 *(lac*$^{+}$ *uvrD*$^{+}$*)* / *ΔlacIZYA Δrep*::*kan ΔuvrD*::*dhfr* | P1.AM1657 x N6661 to Tm$^{r}$ |
| N6671 | pAM407 *(lac*$^{+}$ *uvrD*$^{+}$*)* / *ΔlacIZYA ΔuvrD*::*dhfr Δrep*::*kan tnaA300*::Tn*10 recF143* | P1.JC12334 x N6643 to Tc$^{r}$ |
| N6689 | pAM407 *(lac*$^{+}$ *uvrD*$^{+}$*)* / *ΔlacIZYA Δrep*::*kan ΔuvrD*::*dhfr recA269*::Tn*10* | P1.N3072 x N6669 to Tc$^{r}$ |
| N7120 | *ΔlacIZYA ΔuvrD*::*dhfr Δrep*::*cat* | Plasmid-free derivative of N6644 selected on 56/2 minimal salts agar – this work. |
| N7121 | *ΔlacIZYA ΔuvrD*::*dhfr Δrep*::*kan tnaA300*::Tn*10 recF143* | Plasmid-free derivative of N6671 selected on 56/2 minimal salts agar – this work. |
| N7122 | *ΔlacIZYA ΔuvrD*::*dhfr Δrep*::*cat supX* (=*rpoB*$_{T3713C}$) | Selection of N7120 grown in 56/2 glucose minimal salts medium for growth on LB agar – this work |
| N7124 | pAM407 *(lac*$^{+}$ *uvrD*$^{+}$*)* / *ΔlacIZYA ΔuvrD*::*dhfr Δrep*::*cat supX* (=*rpoB*$_{T3713C}$) | N7122 x pAM407 to Ap$^{r}$ |

| | | |
|---|---|---|
| N7128 | pAM407 *($lac^+$ $uvrD^+$)* / *ΔlacIZYA ΔuvrD*::*dhfr Δrep*::*cat rpoB*$_{T3713C}$ *tnaA300*::Tn*10 recF143* | P1.JC12334 x N7124 to Tc$^r$ |
| N7129 | *ΔlacIZYA Δrep*::*cat ΔrecQ*::*kan ΔuvrD*::*dhfr* | Plasmid-free derivative of N6552 selected on 56/2 minimal salts agar – this work. |
| N7131 | pAM407 *($lac^+$ $uvrD^+$)* / *rpoB*35 ΔlacIZYA* | N5925 x pAM407 to Ap$^r$ |
| N7133 | pAM407 *($lac^+$ $uvrD^+$)* / *ΔlacIZYA ΔuvrD*::*dhfr Δrep*::*cat ΔrecQ*::*kan* | P1.N6499 x N6644 to Km$^r$ |
| N7145 | pAM407 *($lac^+$ $uvrD^+$)* / *rpoB*35 ΔlacIZYA ΔuvrD*::*dhfr* | P1.AM1657 x N7131 to Tm$^r$ |
| N7147 | *ΔlacIZYA ΔuvrD*::*dhfr Δrep*::*cat rpoB*$_{T3713C}$ *tnaA300*::Tn*10 recF143* | Plasmid-free derivative of N7128 selected on LB agar – this work. |
| N7150 | pAM407 *($lac^+$ $uvrD^+$)* / *rpoB*35 ΔlacIZYA ΔuvrD*::*dhfr Δrep*::*cat* | P1. JJC735 x N7145 to Cm$^r$ |
| N7154 | *spoT1 ΔlacIZYA<>aph* | P1.TB12 x N5020 to Km$^r$ |
| N7157 | pAM407 *($lac^+$ $uvrD^+$)* / *spoT1 ΔlacIZYA<>aph* | N7154 x pAM407 to Ap$^r$ |
| N7181 | *ΔlacIZYA ΔuvrD*::*dhfr Δrep*::*cat rpoB*$_{C2489T}$ | Selection of N7120 grown in 56/2 glucose minimal salts medium for growth on LB agar – this work. |
| N7187 | pAM407 *($lac^+$ $uvrD^+$)* / *spoT1 ΔlacIZYA<>aph Δrep*::*cat* | P1.N7147 x N7157 to Cm$^r$ |
| N7205 | pAM407 *($lac^+$ $uvrD^+$)* / *spoT1 ΔlacIZYA<>aph Δrep*::*cat ΔuvrD*::*dhfr* | P1.N7147 x N7187 to Tm$^r$ |
| N7208 | pAM407 *($lac^+$ $uvrD^+$)* / *ΔlacIZYA ΔuvrD*::*dhfr Δrep*::*cat rpoB*$_{C2489T}$ | N7181 x pAM407 to Ap$^r$ |
| N7550 | pAM403 *($lac^+$$rep^+$)* / *ΔlacIZYA Δrep*::*cat ΔuvrD*::*dhfr recJ284*::Tn*10* | P1.N4934 x N6556 to Tc$^r$ |
| N7554 | pAM407 *($lac^+$ $uvrD^+$)* / *ΔlacIZYA Δrep*::*kan ΔuvrD*::*dhfr recJ284*::Tn*10* | P1.N4934 x N6669 to Tc$^r$ |
| N7559 | *ΔlacIZYA Δrep*::*cat ΔuvrD*::*dhfr recJ284*::Tn*10* | Plasmid-free derivative of N7550 selected on 56/2 minimal salts agar – this work. |
| N7639 | pAM407 *($lac^+$ $uvrD^+$)* / *ΔlacIZYA rpoB*$_{G3779A}$ | pAM407 x AM2158 to Ap$^r$ |
| N7643 | pAM407 *($lac^+$ $uvrD^+$)* / *ΔlacIZYA rpoB*$_{G3779A}$ *ΔuvrD*::*dhfr Δrep*::*cat* | P1.N7147 x N7639 to Cm$^r$ and Tm$^r$ |
| PBM004 | pAM407 *($lac^+$ $uvrD^+$)* / *rpoB*35 ΔlacIZYA ΔuvrD*::*dhfr Δrep*::*cat Δara714 leuB::*Tn*10* | P1.LMG194 x N7150 to Tc$^r$ |
| PBM018 | pAM407 *($lac^+$ $uvrD^+$)* / *rpoB*35 ΔlacIZYA Δrep*::*cat Δara714 leuB::*Tn*10 argEC::[apra$^r$ lacO$_{34}$]* | P1.BP41 x PBM004 to Apra$^r$, screened for Cm$^r$ and Tm$^s$ |
| PBM025 | pAM407 *($lac^+$ $uvrD^+$)* / *rpoB*35 ΔlacIZYA Δara714 leuB::*Tn*10* | P1.LMG194 x N7131 to Tc$^r$ |
| PBM029 | pAM407 *($lac^+$ $uvrD^+$)* / *rpoB*35 ΔlacIZYA Δara714 leuB::*Tn*10 argEC::[apra$^r$ lacO$_{34}$]* | P1.PBM018 x PBM025 to Apra$^r$ |
| PBM035 | pAM407 *($lac^+$ $uvrD^+$)* / *rpoB*35 ΔlacIZYA ΔuvrD*::*dhfr Δara714* | P1.N6632 x PBM029 to Tm$^r$ |

| | | |
|---|---|---|
| | *leuB*::Tn*10 argEC::[apra*$^r$ *lacO*$_{34}$*]* | |
| PBM043 | pAM407 *(lac*$^+$ *uvrD*$^+$*)* / *rpoB*35 ΔlacIZYA ΔuvrD*::*dhfr Δrep*::*cat Δara714 leuB*::Tn*10 argEC::[apra*$^r$ *lacO*$_{34}$*]* | P1.N6644 x PBM029 to Cm$^r$ and Tm$^r$ |
| PBM044 | pAM407 *(lac*$^+$ *uvrD*$^+$*)* / *rpoB*35 ΔlacIZYA Δrep*::*cat Δara714 leuB*::Tn*10 argEC::[apra*$^r$ *lacO*$_{34}$*]* | P1.N6577 x PBM029 to Cm$^r$ |
| PM462 | *rpoB*35 ΔlacIZYA Δara714 leuB*::Tn*10 argEC::[apra*$^r$ *lacO*$_{34}$*]* | Plasmid-free derivative of PBM029 selected on LB agar - this work |
| PM463 | *rpoB*35 ΔlacIZYA Δrep*::*cat Δara714 leuB*::Tn*10 argEC::[apra*$^r$ *lacO*$_{34}$*]* | Plasmid-free derivative of PBM044 selected on LB agar - this work |
| PM464 | *rpoB*35 ΔlacIZYA ΔuvrD*::*dhfr Δara714 leuB*::Tn*10 argEC::[apra*$^r$ *lacO*$_{34}$*]* | Plasmid-free derivative of PBM035 selected on LB agar - this work |
| PM465 | *rpoB*35 ΔlacIZYA ΔuvrD*::*dhfr Δrep*::*cat Δara714 leuB*::Tn*10 argEC::[apra*$^r$ *lacO*$_{34}$*]* | Plasmid-free derivative of PBM043 selected on LB agar - this work |
| TB12 | *ΔlacIZYA<>aph* (Km$^r$) | (Bernhardt and de Boer, 2004) |
| TB28 | *ΔlacIZYA<>frt*[g] | (Bernhardt and de Boer, 2004) |

[a]Only the relevant additional genotype of the derivatives is shown

[b]A new deletion allele of *uvrD (uvrD::dhfr)* was made using one-step gene inactivation (Datsenko and Wanner, 2000). The entire coding sequence was deleted and replaced with a sequence encoding resistance to trimethoprim.

## Supplemental References


Al-Deib, A.A., Mahdi, A.A., and Lloyd, R.G. (1996). Modulation of recombination and DNA repair by the RecG and PriA helicases of *Escherichia coli* K-12. J. Bacteriol. *178*, 6782-6789.

Atkinson, J., Guy, C.P., Cadman, C.J., Moolenaar, G.F., Goosen, N., and McGlynn, P. (2009). Stimulation of UvrD helicase by UvrAB. J. Biol. Chem. *284*, 9612-9623.

Bachmann, B.J. (1996). Derivations and genotypes of some mutant derivatives of *Escherichia coli* K-12. In *Escherichia coli* and *Salmonella* cellular and molecular biology, F.C. Neidhardt, R. Curtiss III, J.L. Ingraham, E.C.C. Lin, K.B. Low, B. Magasanik, W.S. Reznikoff, M. Riley, M. Schaechter, and H.E. Umbarger, eds. (Washington, DC: ASM Press), pp. 2460-2488.

Bernhardt, T.G., and de Boer, P.A. (2004). Screening for synthetic lethal mutants in *Escherichia coli* and identification of EnvC (YibP) as a periplasmic septal ring factor with murein hydrolase activity. Mol. Microbiol. *52*, 1255-1269.

Blinkova, A., Hervas, C., Stukenberg, P.T., Onrust, R., O'Donnell, M.E., and Walker, J.R. (1993). The Escherichia coli DNA polymerase III holoenzyme contains both products of the dnaX gene, tau and gamma, but only tau is essential. J. Bacteriol. *175*, 6018-6027.

Brendza, K.M., Cheng, W., Fischer, C.J., Chesnik, M.A., Niedziela-Majka, A., and Lohman, T.M. (2005). Autoinhibition of *Escherichia coli* Rep monomer helicase activity by its 2B subdomain. Proc. Natl. Acad. Sci. U S A *102*, 10076-10081.

Cadman, C.J., Matson, S.W., and McGlynn, P. (2006). Unwinding of forked DNA structures by UvrD. J. Mol. Biol. *362*, 18-25.

Cheng, W., Brendza, K.M., Gauss, G.H., Korolev, S., Waksman, G., and Lohman, T.M. (2002). The 2B domain of the *Escherichia coli* Rep protein is not required for DNA helicase activity. Proc. Natl. Acad. Sci. USA *99*, 16006-16011.

Cherepanov, P.P., and Wackernagel, W. (1995). Gene disruption in *Escherichia coli*: $Tc^R$ and $Km^R$ cassettes with the option of Flp-catalyzed excision of the antibiotic-resistance determinant. Gene *158*, 9-14.

Datsenko, K.A., and Wanner, B.L. (2000). One-step inactivation of chromosomal genes in *Escherichia coli* K-12 using PCR products. Proc. Natl. Acad. Sci. U S A *97*, 6640-6645.

Guzman, L.M., Belin, D., Carson, M.J., and Beckwith, J. (1995). Tight regulation, modulation, and high-level expression by vectors containing the arabinose $P_{BAD}$ promoter. J. Bacteriol. *177*, 4121-4130.

Heller, R.C., and Marians, K.J. (2005). Unwinding of the nascent lagging strand by Rep and PriA enables the direct restart of stalled replication forks. J. Biol. Chem. *280*, 34143-34151.

Jaktaji, R.P., and Lloyd, R.G. (2003). PriA supports two distinct pathways for replication restart in UV-irradiated Escherichia coli cells. Mol. Microbiol. *47*, 1091-1100.

Kim, D.R., and McHenry, C.S. (1996). In vivo assembly of overproduced DNA polymerase III. Overproduction, purification, and characterization of the alpha, alpha-epsilon, and alpha-epsilon-theta subunits. J. Biol. Chem. *271*, 20681-20689.

King, K., Benkovic, S.J., and Modrich, P. (1989). Glu-111 is required for activation of the DNA cleavage center of *Eco*RI endonuclease. J. Biol. Chem. *264*, 11807-11815.

Kodaira, M., Biswas, S.B., and Kornberg, A. (1983). The *dnaX* gene encodes the DNA polymerase III holoenzyme tau subunit, precursor of the gamma subunit, the *dnaZ* gene product. Mol. Gen. Genet. *192*, 80-86.

Lloyd, R.G., Buckman, C., and Benson, F.E. (1987). Genetic analysis of conjugational recombination in *Escherichia coli* K12 strains deficient in RecBCD enzyme. J. Gen. Microbiol. *133*, 2531-2538.

Mahdi, A.A., Briggs, G.S., Sharples, G.J., Wen, Q., and Lloyd, R.G. (2003). A model for dsDNA translocation revealed by a structural motif common to RecG and Mfd proteins. EMBO J. *22*, 724-734.

Mahdi, A.A., Buckman, C., Harris, L., and Lloyd, R.G. (2006). Rep and PriA helicase activities prevent RecA from provoking unnecessary recombination during replication fork repair. Genes Dev. *20*, 2135-2147.

Marians, K.J. (1995). ϕX174-type primosomal proteins: purification and assay. Methods Enzymol. *262*, 507-521.

Onrust, R., Finkelstein, J., Turner, J., Naktinis, V., and O'Donnell, M. (1995). Assembly of a chromosomal replication machine: two DNA polymerases, a clamp loader, and sliding clamps in one holoenzyme particle. III. Interface between two polymerases and the clamp loader. J. Biol. Chem. *270*, 13366-13377.

Payne, B.T., van Knippenberg, I.C., Bell, H., Filipe, S.R., Sherratt, D.J., and McGlynn, P. (2006). Replication fork blockage by transcription factor-DNA complexes in *Escherichia coli*. Nucleic Acids Res. *34*, 5194-5202.

Reece, K.S., and Phillips, G.J. (1995). New plasmids carrying antibiotic-resistance cassettes. Gene *165*, 141-142.

Saikrishnan, K., Griffiths, S.P., Cook, N., Court, R., and Wigley, D.B. (2008). DNA binding to RecD: role of the 1B domain in SF1B helicase activity. EMBO J. *27*, 2222-2229.

Studwell-Vaughan, P.S., and O'Donnell, M. (1993). DNA polymerase III accessory proteins. V. Theta encoded by holE. J. Biol. Chem. *268*, 11785-11791.
